# The effects of core stability training on swimming performance in youth swimmers: a systematic review and meta-analysis

**DOI:** 10.1186/s13102-025-01366-1

**Published:** 2025-11-11

**Authors:** Shunfang Liu, Jinming Dai, Pengpeng Gou, Menglong Lin

**Affiliations:** 1 School of Physical Education and Sports Science, South China Normal University, Guangzhou, China; 2 Program in Global Exercise Science, Arts & Sports College, Inha University, Incheon, South Korea; 3 Faculty of Sport and Leisure, Guangdong Ocean University, Zhanjiang, China

**Keywords:** Core stability training, Swimming performance, Meta-analysis, Dose effect

## Abstract

**Background:**

The effectiveness of core stability training, a key component of physical conditioning, on swimming performance remains a topic of debate.

**Methods:**

This study performed a systematic review and meta-analysis to evaluate the overall effect of core stability training on swimming performance and to examine the moderating effects of gender, stroke type, and training dose. A total of 16 controlled trials (*n* = 438) were identified through a systematic search of eight electronic databases and additional sources, following PRISMA guidelines. Methodological quality was assessed using the PEDro scale. The standardized mean difference was used to pool effect sizes under a random-effects model.

**Results:**

After excluding two studies that contributed substantial heterogeneity, core stability training demonstrated a moderate and statistically significant effect on swimming performance (SMD = -0.71, 95% CI: -0.91 to -0.51, *p* < 0.00001), with low residual heterogeneity (I^2^ = 21%). Subgroup analyses indicated that core stability training was most effective for the 50 m sprint and backstroke events. The most effective training parameters included an 8-week intervention, ≤ 3 sessions per week, and session durations of > 30 to ≤ 60 min. Additionally, male swimmers showed slightly greater improvements than female swimmers.

**Conclusion:**

In conclusion, core stability training appears effective in enhancing short-distance swimming performance. However, training programs should be tailored to stroke-specific biomechanics, and further studies involving adult populations and long-term interventions are needed to confirm its sustained benefits.

**Supplementary Information:**

The online version contains supplementary material available at 10.1186/s13102-025-01366-1.

## Introduction

‘Core stability’ (CS) is defined as the ability to control the position and movement of the trunk relative to the pelvis [1]. Its function relies on the core musculature, including the latissimus dorsi, abdominals, pelvic floor, diaphragm, hip, and gluteal muscles [1, 2]. These muscles serve as 'power hubs' linking the upper and lower extremities, playing a crucial role in maintaining spinal stability and optimizing force transmission efficiency [2]. Currently, core stability training (CST), which targets the core muscle groups, is being investigated in both clinical rehabilitation and athletic settings. CST was initially used in medical rehabilitation, and its effectiveness has been demonstrated in numerous clinical studies [3–7]. In recent years, with the advancement of sports science, CST has been increasingly applied in sports training due to its role in trunk stability, power chain transmission efficiency, and body posture control [8]. Numerous empirical studies have shown that CST is beneficial for cyclical sports [9–11], and also effective in competitive sports such as basketball, volleyball, and football [12–16]. Some meta-analyses have shown that although CST improves certain performance variables [17, 18], further research is needed to explore its effectiveness across different sporting disciplines and assess its specificity [19].

Swimming, a full-body, coordinated, cyclical activity, relies on generating propulsive force and reducing resistance in the water [20]. Maintaining a streamlined body posture and optimal dynamic-static balance can significantly reduce water resistance and enhance athletic performance, both of which depend on core muscle stability [21]. For instance, breaststroke requires a high degree of trunk coordination and dynamic balance during the leg kick and arm stroke phases [22], whereas freestyle requires core control to minimize lateral sway and maintain a streamlined posture [23]. Butterfly and backstroke also demand significant core muscle involvement. The wave-like body motion of the butterfly stroke demands a high degree of synergy among trunk muscles [24], while backstroke relies on core muscles to maintain body balance against water resistance [25].

Although several studies have explored the potential value of CST for swimming, the existing evidence remains contradictory regarding its actual benefits. Some studies have demonstrated that CST significantly improves swimming performance [26–30], while others have found no significant improvements [31–33]. This potential bias may arise from inconsistencies such as a lack of randomisation, small sample sizes, and variations in CST protocols, which limit the comparability of results. For instance, Eskiyecek and Karpinski et al. employed randomised allocation methods [27, 28], enhancing the credibility of their experimental evidence. In contrast, Hepsert et al. used an uncontrolled pre–post test design [33]. Additionally, Weston et al. implemented a 12-week core training program involving 20 participants, with sessions held three times per week and lasting approximately 30 min each. The exercises targeted the lumbopelvic region and extended to the scapular area [26]. Similarly, Khiyami et al. implemented a six-week core training program for 18 participants, conducted three times per week with 60-min sessions. The exercises covered regions from the shoulder to the lumbopelvic area [30]. Therefore, it is necessary to synthesise existing studies using meta-analytic techniques to enhance the scientific validity of CST.

In addition, previous review studies have primarily focused on a single stroke (e.g., freestyle) and have not systematically examined the influence of other moderating variables, such as stroke type and dose effect. For instance, two meta-analyses reported positive effects of CST on 50 m freestyle performance [34, 35], and one of them also examined its impact on 100 m freestyle [35]. However, both studies were limited to these distances and did not explore the effects of CST on other strokes (e.g., breaststroke, butterfly) or the dose–response relationship.

Therefore, this systematic review and meta-analysis aims to incorporate as much primary evidence as possible and address the following questions: (1) What is the overall effect of CST on swimming performance? (2) How do variables such as gender, stroke, and training period modulate the effect of CST? The study results will provide an evidence-based foundation for optimizing swimming fitness training programs and address gaps in the existing literature regarding the analysis of moderating variables and multi-stroke comparisons.

## Methods

This study adhered to the Preferred Reporting Items for Systematic Reviews and Meta-Analyses (PRISMA) guidelines [36] and was registered with PROSPERO (registration number: CRD42025641237).

### Search strategy

To gather as many original studies as possible, an independent researcher systematically searched eight databases—Web of Science, PubMed, SPORTDiscus, ScienceDirect, Scopus, Cochrane Library, Embase, and ProQuest—using Boolean operators and the following search terms: ("Core Training" OR "Core Strength Training" OR "Core Stability Training" OR "Core Endurance Training" OR "Core Muscles Training" OR "Core Exercises") AND ("Swimming Exercise" OR "Swimming Performance" OR Swimming). No restrictions were imposed on the search process (e.g., date, language, or publication type), and the final search was conducted on 28 February 2025. Additionally, we screened the included literature and references from relevant review articles to identify further studies eligible for inclusion in this systematic review. Finally, we searched websites such as Google Scholar and ResearchGate for any missing literature. Two independent reviewers screened the retrieved literature based on titles and abstracts. After the initial screening, the full text was reviewed according to the selection criteria to filter studies for inclusion in this systematic review. If disagreements arose between the two reviewers during this process, a third researcher was involved in the discussion to reach a consensus.

### Selection and exclusion criteria

Included articles had to meet the following criteria: (1) the subjects were healthy individuals; (2) the study design was a randomized controlled trial or a controlled clinical trial; (3) the intervention administered to the experimental group was CST, which specifically included strength, endurance, or stability-focused exercises targeting the core muscle groups of the trunk (e.g., abdominal, back, gluteal, and hip muscles). These exercises share the common characteristic of emphasizing trunk-centered training, promoting coordinated muscle activation, with the primary goal of improving core stability. The control group received the same routine training as the experimental group but without the additional core stability component. This ensured that any between-group differences could be attributed solely to the implementation of CST; (4) the outcome metrics included at least one swimming performance measure and data for calculating effect sizes; (5) the full text was available for download..

Articles were excluded if they met one or more of the following criteria: (1) the subjects were animals or unhealthy populations; (2) the study design was an uncontrolled clinical trial; (3) the study did not include swimming performance outcomes or lacked sufficient data to calculate effect sizes; (4) the full text was not available for download..

### Study quality

Since nearly all articles in this study were randomized controlled trials, two independent reviewers evaluated the risk of bias and methodological quality of eligible articles using the Physiotherapy Evidence Database (PEDro) scale [37]. The scale evaluates 11 items: inclusion criteria and source, random allocation, concealed allocation, baseline similarity, subject blinding, therapist blinding, assessor blinding, completeness of follow-up, intention-to-treat analysis, between-group statistical comparisons, and point measurements and variability. Each item is rated as 'yes' or 'no', with a maximum score of 10 (excluding the inclusion criteria and source items) [38]. Complete blinding of participants, therapists, and assessors during physical training interventions is generally infeasible. Therefore, following Saeterbakken et al., we excluded items 5, 6, and 7 from the PEDro scale, reducing the maximum score to 7 [18]. Based on previous systematic evaluations [18, 39], study quality was interpreted as follows: scores of 6–7 were considered ‘excellent’, 5 as ‘good’, 4 as ‘moderate’, and 0–3 as ‘poor’. If two independent reviewers disagreed on the rating of an article, a third researcher participated in the discussion to reach a consensus.

### Data extraction

The study coded each article based on the following variables: first author, sample size, gender, age, swimming experience, experimental and control group treatments, dose parameters (weeks, frequency, and duration of a single training session), and swimming performance indicators. Data for this study were independently extracted by two reviewers. Discrepancies were resolved through discussion, and a third reviewer was consulted to arbitrate any unresolved conflicts when necessary. Although inter-rater reliability statistics were not calculated, all discrepancies were resolved prior to data synthesis to ensure consistency and objectivity. The experimental group treatment categories included core training, core strength training, CST, Swiss ball training, and other interventions targeting core muscle groups. Swimming performance indicators refer specifically to the time required to complete a given swimming event—for example, the duration to finish a 50 m freestyle race under standard conditions. In cases where a study included three or more groups (e.g., two experimental groups and one control group, or an experimental group, a control group, and a blank group), the group receiving CST was designated as the experimental group. The group receiving an identical protocol but without CST was designated as the control group. All other groups were excluded from the analysis.

### Statistical analysis

In this study, Review Manager (RevMan) version 5.3 was used to analyze the extracted data (sample size, mean, and standard deviation) for overall effect analysis and bias testing, while Stata version 15.0 was used for subgroup and influence analyses when necessary. Due to variations in CST programmes, study populations, and outcome measures, a random-effects model was used for the analysis. Heterogeneity, referring to the variation in main effects across studies, was assessed using the I^2^ statistic [40, 41]. I^2^ values of 25%, 50%, and 75% correspond to low, medium, and high heterogeneity, respectively [41]. This study combined several original studies that included data from different swimming strokes and evaluation criteria, using standardized mean difference (SMD) for analysis. In addition, subgroup results were synthesized using SMD. A *p*-value < 0.05 indicates statistical significance. SMD values were categorized as tiny (SMD < 0.2), small (0.2 ≤ SMD < 0.5), medium (0.5 ≤ SMD < 0.8), and large (SMD ≥ 0.8) [42].

To explore the influence of potential moderators on the effects of CST, we conducted subgroup analyses. To ensure a comprehensive evaluation of swimming performance, studies were stratified according to race events, distances, and stroke types. The subgrouping criteria included three main classification variables: swimming event (25 m Freestyle vs. 50 m Freestyle vs. 50 m Butterfly vs. 50 m Backstroke vs. 50 m Breaststroke vs. 100 m medley stroke vs. 100 m Backstroke vs. 100 m Freestyle), swimming distance (25 m vs. 50 m vs. 100 m), stroke type (Freestyle vs. Breaststroke vs. Backstroke vs. Butterfly vs. medley stroke). Each individual swimming performance outcome was classified into the corresponding category based on its specific event designation.

In addition, we conducted subgroup analyses based on the following moderator variables:

Sex (male vs. female): Only studies involving participants of a single sex were included. The male group comprised all studies with male-only participants, while the female group included those with female-only participants. Studies with mixed-sex participants were excluded from this subgroup analysis to avoid potential confounding effects. The results of mixed-sex studies were included only in the overall effect analysis.

Training period (6 vs. 8 weeks): Sixteen studies reported either a 6-week or 8-week intervention period and were therefore classified accordingly.

Frequency (≤ 3 vs. > 3 sessions/week): Among the 16 studies, training frequencies were reported as follows: 2 sessions/week (1 study), 3 sessions/week (11 studies), 4 sessions/week (1 study), and 5 sessions/week (2 studies). Given the variability, a threshold of 3 sessions/week was used to define two subgroups: ≤ 3 sessions/week and > 3 sessions/week, to examine the impact of training frequency on CST outcomes.

Session duration (≤ 30 min vs. > 30 to ≤ 60 min): Of the 16 studies, 6 did not report session duration. The remaining studies reported durations of 20 min, 25 min, 30–60 min, 35–45 min, and similar ranges. Therefore, 30 min was selected as the threshold, categorizing sessions as ≤ 30 min (including 30 min) versus > 30 to ≤ 60 min.

If a subgroup contained fewer than three studies (k < 3), effect sizes were reported descriptively due to the high uncertainty, and results were presented for reference only.

## Results

### Study characteristics

**Fig. 1:**
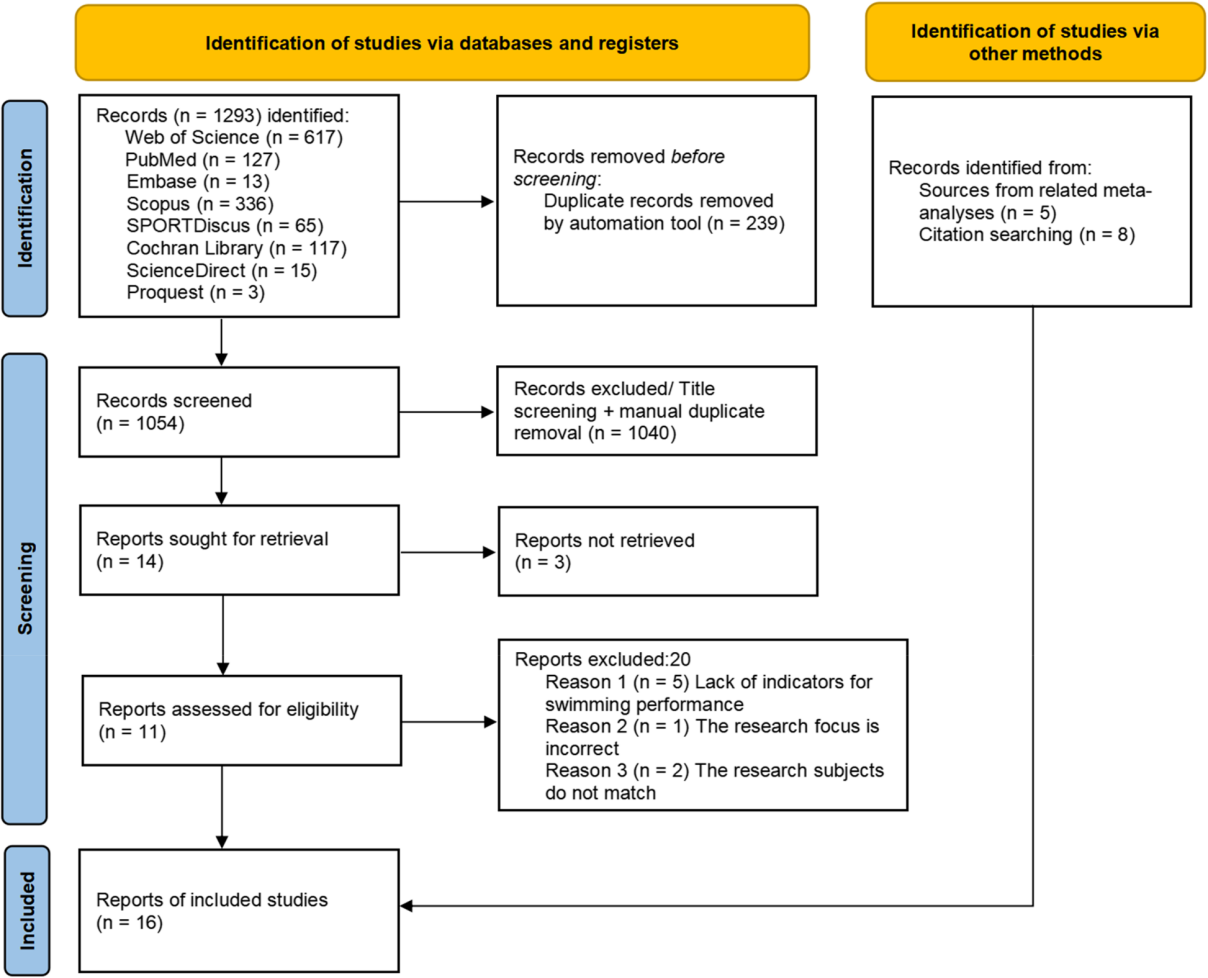
Flow diagram of article retrieval and screening for inclusion in the meta-analysis

The flowchart illustrates the entire systematic retrieval process for this study (Fig. 1). The database search identified 1,293 potential articles. After independent screening by two reviewers with no disagreements, three articles [27, 28, 30] met the inclusion criteria, and an additional 13 articles [21, 32, 43–53] were identified from other sources, yielding a total of 16 included studies. The 16 studies initially included 461 participants. After excluding 6 dropouts [30] and 17 irrelevant comparison groups [50, 52], the final pooled sample consisted of 438 participants (CST group = 219, control group = 219). Basic information about the articles is as follows: in terms of gender, 10 studies included males, 2 included females, 3 included both genders, and 1 study did not report gender. In terms of age, only one study [28] included adult participants, while the remaining 15 studies involved adolescents. Regarding swimming experience, all 438 subjects had some swimming experience and were not beginners. Regarding swimming performance, eight studies reported 25 m/50 m/100 m freestyle, three studies reported 50 m/100 m backstroke, one study reported only 50 m butterfly [46], one study reported 100 m medley [43], and three studies reported 50 m freestyle, breaststroke, backstroke, and butterfly. Regarding dose parameters, 12 studies reported training weeks, frequency per week, and session duration, four studies reported training weeks and frequency, but did not report session duration.

The quality of the 16 articles was assessed using the PEDro scale, with the results shown in Table 1. The maximum possible score was 7 (see 2.3 for detailed explanation), with the median quality score being 5 (95% confidence interval (CI) 4.8–5.5). Articles scoring 5 or higher were categorized as having good methodological quality with a low risk of bias. A total of 16 articles were included in this study, with 15 scoring ≥ 5 and 1 scoring 4. Regarding quality, 14 articles were rated as 'excellent', 1 as 'good', and 1 as 'moderate'.

**Table 1:** Basic characteristics of included studies in the meta-analysis (*n* = 16). M mean, SD standard deviation

Study	Number of subjects	Sex	Age (M ± SD)	Swimming experience (M ± SD)	Experimental treatment/control treatment	Dose parameters (weeks/frequency/session duration)	Swimming performance index	PEDro score
Dnyanesh Patil et al. [21]	60	CST: 19 males, 11 females CON: 19 males, 11 females	CST: male = 14.7 ± 1.29 years female = 13.4 ± 1.50 years CON: male = 14.7 ± 1.29 years female = 13.4 ± 1.50 years	at least 2 years	core training/Non-core training	6 weeks/3 session per week/30–60 min	50 m Freestyle	7
Canan Gülbin Eskiyecek et al. [27]	24	24 males	CST: 11.25 ± 0.75 years CON: 10.42 ± 0.51 years	CST: 28 ± 2.52 months CON: 28.50 ± 3.03 months	core exercise/Non-core training	8 weeks/3 session per week/not report	50 m Freestyle 50 m Backstroke 50 m Butterfly 50 m Breaststroke	6
Jakub Karpiński et al. [28]	16	16 males	CST: 20.2 ± 1.17 years CON: 20.0 ± 1.9 years	At least 10 years	core exercises/Non-core training	6 weeks/3 session per week/25 min	50 m Freestyle	6
Ahmad Khiyami et al. [30]	18	18 males	CST: 13 ± 2 years CON: 13.11 ± 2.6 years	CST: 2.8 ± 0.4 years CON: 2.9 ± 0.7 years	core training/Non-core training	6 weeks/3 session per week/60 min	50 m Freestyle	6
Kwok Wan Yu [32]	32	CST: 8 males, 8 females CON: 10 males, 6 females	CST: male = 14.87 ± 1.64 years female = 14.35 ± 1.06 years CON: male = 17.40 ± 3.92 years female = 15.33 ± 2.16 years	CST: male = 6.38 ± 0.11 years female = 7.00 ± 1.69 years CON: male = 9.90 ± 2.85 years female = 7.00 ± 1.10 years	core training/Non-core training	8 weeks/2 session per week/35–45 min	50 m Freestyle	6
Kaan Özdoğru [43]	60	60 males	CST: 10.20 ± 1.27 years CON: 10.10 ± 1.274 years	At least 2 years	dynamic core training/Non-core training	8 weeks/5 session per week/20 min	100 m medley stroke	7
Ahmet Gönener et al. [44]	24	24 males	CST: 14.08 ± 0.79 years CON: 13.91 ± 0.79 years	CST: 4.08 ± 1.44 years CON: 3.83 ± 1.26 years	core training/Non-core training	8 weeks/3 session per week/not report	100 m Backstroke	7
P. Sedaghati et al. [45]	24	24 females	CST: 14.08 ± 1.08 years CON: 14.00 ± 1.27 years	CST: 7.83 ± 1.19 years CON: 7.08 ± 1.67 years	core stability training/Non-core training	8 weeks/3 session per week/40–50 min	50 m Freestyle 100 m Freestyle	7
Ika Novitaria Marani [46]	30	16 males 14 females	10–13 years	Athletes with four strokes	core stability exercise/Non-core training	6 weeks/3 session per week/not report	50 m Butterfly	6
Mostafa Zarei et al. [47]	30	30 males	CST: 15 ± 1 years CON: 15 ± 0.05 years	at least 3 years	core stability training/Non-core training	8 weeks/3 session per week/30–50 min	50 m Freestyle 50 m Backstroke 50 m Butterfly 50 m Breaststroke	7
Mucahit SARIKAYA et al. [48]	20	20 males	CST: 11.8 ± 0.78 years CON: 12.3 ± 0.82 years	Athletes from the swimming club	core training/Non-core training	8 weeks/3 session per week/60 min	50 m Backstroke	7
Yaşar Mayda et al. [49]	16	16 males	16.5 ± 1.37 years	at least 5 years	core training/Non-core training	8 weeks/3 session per week/60 min	50 m Freestyle 50 m Backstroke 50 m Butterfly 50 m Breaststroke	6
Mine Gül et al. [50]	14	14 males	CST: 11.57 ± 1.272 years CON: 11.43 ± 1.272 years	swimmers	core stabilization training/Non-core training	8 weeks/3 session per week/not report	50 Backstroke	6
Yıldırım Gökhan GENCER [51]	24	24 females	CST: 10.58 ± 1.31 years CON: 10.75 ± 1.29 years	at least 3 years	core exercise/Non-core training	8 weeks/5 session per week/20 min	25 m Freestyle 50 m Freestyle	5
Songül KURT et al. [52]	22	not report	12–15 years	swimmers	core training/Non-core training	8 weeks/4 session per week/30 min	50 m Freestyle 100 m Freestyle	4
M Darchini et al. [53]	24	24 males	12.60 ± 1.60 years	swimmers	core stabilization training/Non-core training	6 weeks/3 session per week/45–50 min	100 m Freestyle	6

### Holistic analysis

**Fig. 2:**
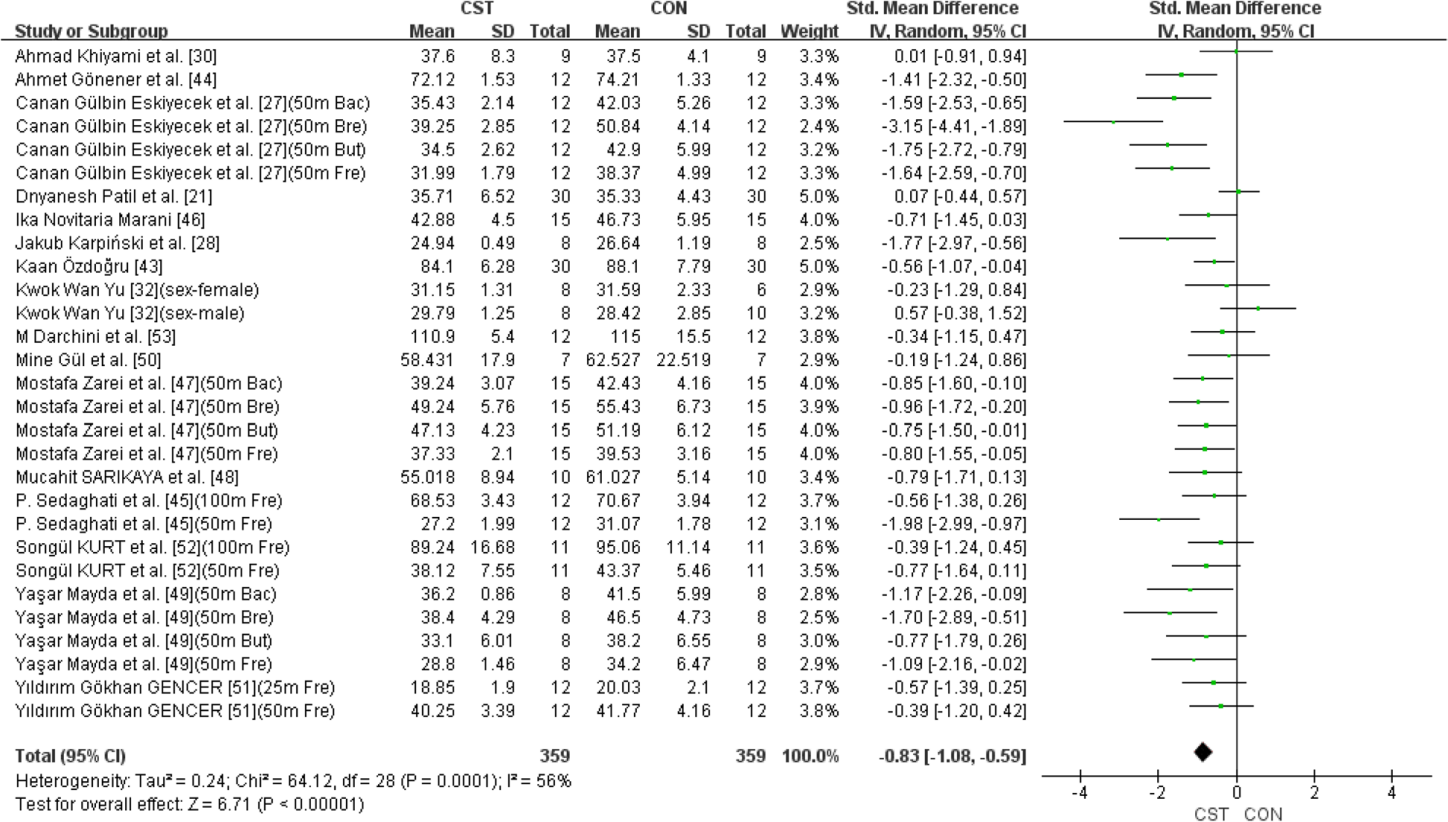
Forest plot showing the overall effect of CST on swimming performance. Mean mean, SD standard deviation, Total sample size, Std. Mean Difference standardized mean difference, CI confidence interval, df degrees of freedom, IV inverse variance, Random random effects model, CST CST group, CON control group, Fre freestyle, But butterfly, Bac Backstroke, Bre Breaststroke

**Fig. 3:**
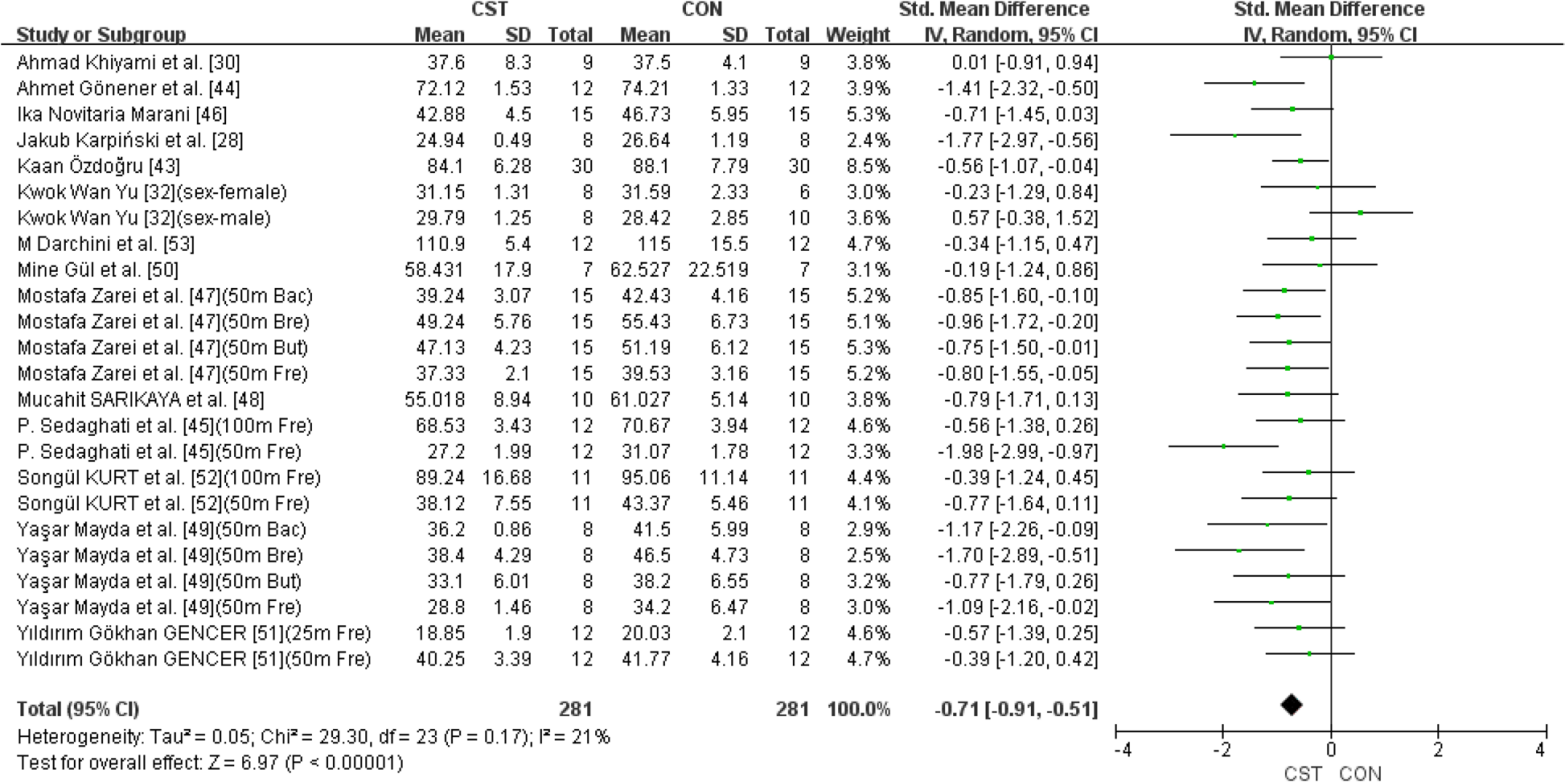
Forest plot after removing heterogeneous data. Mean mean, SD standard deviation, Total sample size, Std. Mean Difference standardized mean difference, CI confidence interval, df degrees of freedom, IV inverse variance, Random random effects model, CST CST group, CON control group, Fre freestyle, But butterfly, Bac Backstroke, Bre Breaststroke

**Fig. 4:**
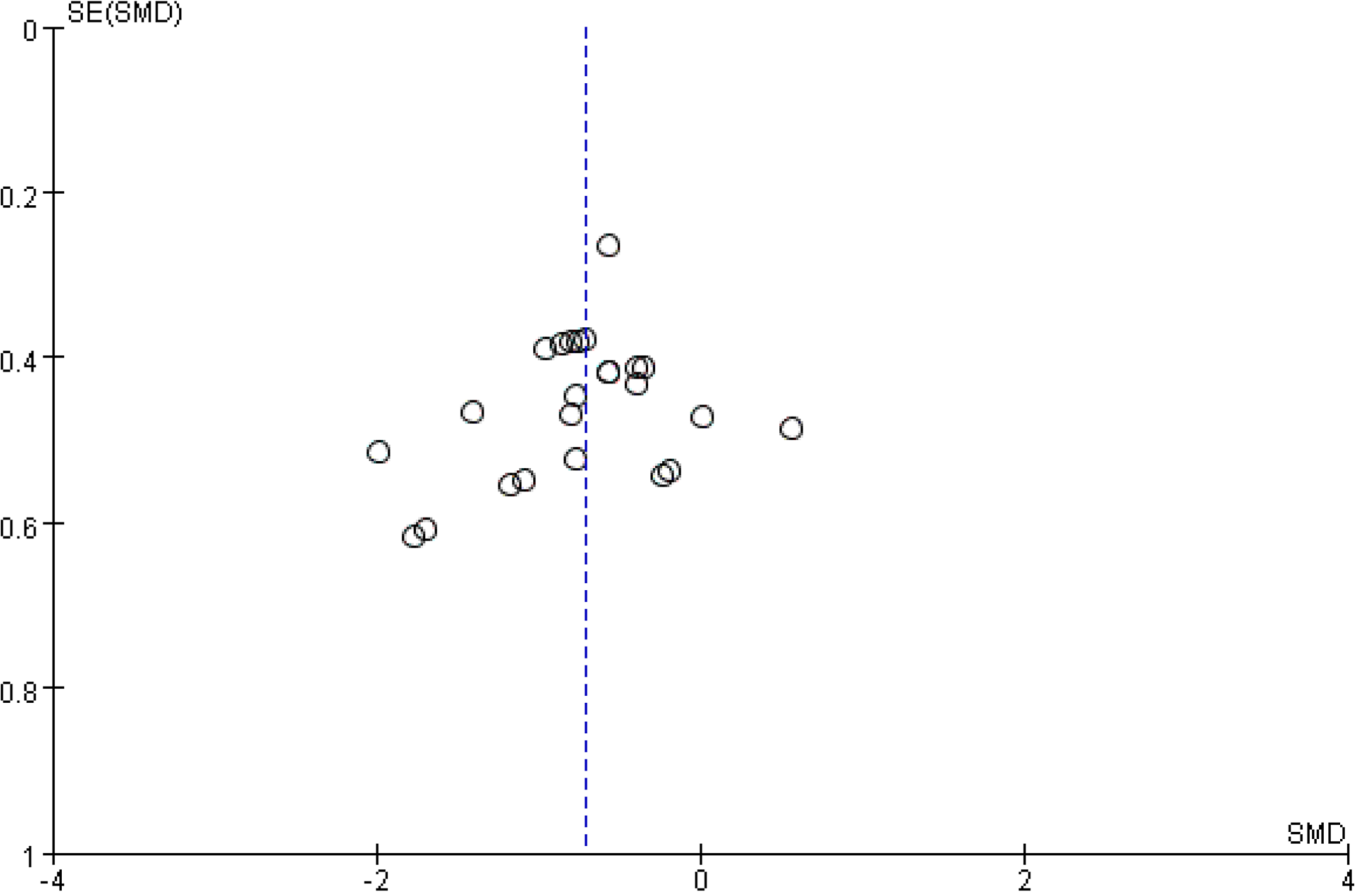
Funnel plot indicating publication bias among included studies

A total of 29 swimming performance indicators from 16 studies were included in the analysis to assess the combined effect of CST on swimming performance. Given that nearly all participants in the included studies were adolescents, the applicability of the results should be interpreted with caution. The pooled SMD was −0.83 (95% CI: −1.08 to −0.59, *p* < 0.00001; I^2^ = 56%) (Fig. 2). This study used time as a performance indicator, where a shorter time indicates better swimming performance. Therefore, the results indicate that CST has a significant and large effect on swimming performance (SMD ≥ 0.8), accompanied by substantial heterogeneity (I^2^ > 50%). To explore the source of heterogeneity, an influence analysis was conducted, revealing that the studies by Dnyanesh Patil et al. [21] and Canan Gülbin Eskiyecek et al. [27] contributed substantially to the overall heterogeneity. Following internal discussions among the three reviewers and considering baseline data inconsistencies in the two studies prior to intervention, we unanimously agreed to exclude them. After excluding these two studies, heterogeneity was reduced from high to low (I^2^ = 21% < 25%), and the updated effect size was SMD = −0.71 (95% CI: −0.91 to −0.51, *p* < 0.00001) (Fig. 3). Publication bias was subsequently assessed using funnel plots and Egger’s test. The funnel plots appeared relatively symmetrical (Fig. 4), and Egger’s test was not statistically significant (*p* = 0.219 > 0.05), indicating no substantial publication bias among the included studies.

### Subgroup analysis

Table 2 presents the effects of each subgroup on CST outcomes for swimming performance. Regarding gender, CST had a greater and significant effect on male swimmers (SMD = − 0.734, *p* = 0.000). Regarding swim parameters, CST showed the largest and significant effects on 50 m backstroke (SMD = − 0.765, *p* = 0.001), backstroke overall (SMD = − 0.896, *p* = 0.000), and 50 m events (SMD = − 0.759, *p* = 0.000). Although a greater effect size was observed for breaststroke, this subgroup included only two studies (k < 3), limiting the statistical reliability of the result. Therefore, this finding is presented as descriptive only. Regarding dosage parameters, treatment for 8 weeks (SMD = − 0.732, *p* = 0.000), ≤ 3 sessions per week (SMD = − 0.778, *p* = 0.000), and a single training session duration of > 30 to ≤ 60 min (SMD = − 0.731, *p* = 0.000) were the most effective combinations, showing the largest and significant effect sizes for these parameters.

**Table 2:** Subgroup analysis of the effects of CST

Subgroup	Category	Heterogeneity test (I^2^)	Effect size [95%CI] (SMD)	Two-tailed test		Number of studies (Swimming performance index) included
				**Z**	**P**	
Sex	**male**	**28.6%**	**−0.734 [−0.993, −0.475]**	**5.556**	**0.000**	**10 (16)**
	female	48.3%	−0.718 [−1.274, −0.163]	2.533	0.011	3 (5)
Swimming event	50 m Fre	60.2%	−0.681 [−1.181, −0.182]	2.672	0.008	8 (9)
	50 m But	0.0%	−0.738 [−1.206, −0.271]	3.094	0.002	3 (3)
	**50 m Bac**	**0.0%**	**−0.765 [−1.226, −0.304]**	**3.254**	**0.001**	**4 (4)**
	50 m Bre^a^	——	−1.180 [−1.839, −0.522]	——	——	2 (2)
	100 m Fre	0.0%	−0.432 [−0.907, 0.044]	1.780	0.075	3 (3)
Stroke type	Freestyle	42.4%	−0.599 [−0.929, −0.269]	3.558	0.000	9 (13)
	Butterfly	0.0%	−0.738 [−1.206, −0.271]	3.094	0.002	3 (3)
	**Backstroke**	**0.0%**	**−0.896 [−1.307, −0.485]**	**4.270**	**0.000**	**5 (5)**
	Breaststroke^a^	——	−1.180 [−1.839, −0.522]	——	——	2 (2)
Swimming distance	**50 m**	**32.0%**	**−0.759 [−1.020, −0.497]**	**5.685**	**0.000**	**11** (18)
	100 m	0%	−0.608 [−0.934, −0.281]	3.647	0.000	5 (5)
Weeks	6	47.9%	−0.617 [−1.238, 0.004]	1.947	0.052	4 (4)
	**8**	**18.0%**	**−0.732 [−0.945, −0.519]**	**6.739**	**0.000**	**10** (20)
Frequency	** ≤ 3**	**34.3%**	**−0.778 [−1.036, −0.520]**	**5.912**	**0.000**	**11** (19)
	> 3	0.0%	−0.537 [−0.862, −0.212]	3.240	0.001	3 (5)
Session duration	≤ 30 min	0.0%	−0.620 [−0.934, −0.306]	3.874	0.000	4 (6)
	** > 30to ≤ 60 min**	**35.0%**	**−0.731 [−1.020, −0.442]**	**4.951**	**0.000**	**7** (15)

*Fre* Freestyle, *But* Butterfly, *Bac* Backstroke, *Bre* Breaststroke, ^a^the number of included studies was less than 3

## Discussion

### Main analysis

This meta-analysis aimed to investigate the effect of CST on swimming performance. The results revealed a moderate and statistically significant effect of CST compared to the control group, with a pooled SMD of −0.71 (95% CI: −0.91 to −0.51, *p* < 0.00001), and low heterogeneity (I^2^ = 21%). No evidence of publication bias was observed (Egger’s test: *p* = 0.219), further supporting the robustness of the findings. These findings suggest that CST is likely associated with improved swimming performance; however, no study to date has established a direct causal relationship. Indeed, as the subjects included were almost exclusively children and adolescents, adolescents have a wide range of overall development, physical differences, growth rates, and physical skills [54], and the increase in muscle mass, strength, and cardiorespiratory endurance during adolescence is greater than at any other age [55], which may contribute to heterogeneity and limit the applicability of the study results. Moreover, variation in training protocols (e.g., periodization, intensity, supervision) may also account for the heterogeneity observed across studies [56]. For example, some studies implemented 8-week interventions (SMD = − 0.732), while others used only 6-week programs (SMD = − 0.617). A shorter training duration may limit structural adaptations, such as muscle fiber thickening [57]. Although the number of included studies was relatively small, their methodological quality was generally high. Among the 14 studies ultimately included in the analysis, 12 were rated as ‘excellent’ (score ≥ 6) [28, 30, 32, 43–50, 53], one as ‘good’ (score = 5) [51], and one as ‘moderate’ (score = 4) [52]. Although all included studies explicitly applied CST as defined by the inclusion criteria of this review, potential conceptual heterogeneity in training content and implementation still exists. Future studies should clearly define the specific components and protocols of CST interventions and standardize terminology to improve reproducibility and comparability across studies.

To the best of the authors' knowledge, this is the third report on the effect of CST on swimming performance. The first report [34] examined male and female 50 m freestyle performance and found that the effect size was greater for females than for males. However, it included only seven articles, with five and two swimming performance indicators used to analyze male and female effect sizes, respectively. Due to the small number of articles, the results may be influenced by chance. The second report [35] included 11 articles and found that CST improved 50 m and 100 m freestyle times, reducing them by 1.06 s and 2.26 s, respectively. However, the evidence quality for the 100 m freestyle was lower. In contrast, the present study included 15 papers, which combined various swimming strokes, making the results potentially more reliable. Although the interaction effect of gender and stroke was not directly analyzed in this study, Table 2 shows that the effect size was slightly higher for male swimmers (SMD = − 0.734, *p* = 0.000) than for female swimmers (SMD = − 0.718, *p* = 0.011). This differs from the results of the first report [34], and this discrepancy is likely due to differences in specific swim strokes. Combining stroke data, it can be hypothesized that male dominance in breaststroke and butterfly may be related to their superior muscle coordination and dynamic balance. For example, Yaprak et al. found that gender affects dynamic balance parameters, with males showing greater dynamic balance after 8 weeks of core training [58]. However, the technical finesse of female swimmers may have offset some of the strength differences. Some differences were observed between gender subgroups, but it was small. Moreover, the female subgroup included only five swimming performance outcomes in three studies, which substantially limited the reliability of the subgroup analysis. And, most participants in the included studies were children and adolescents, whose muscle development and physical performance may undergo rapid changes and fluctuations due to the combined effects of training and natural growth [59], highlighting the need for future studies to investigate the interaction between gender and swimming style.

### Subgroup analysis related to swimming variable

This study investigated the moderating effect of swimming-specific parameters (stroke and distance) on the impact of CST through subgroup analyses. The results revealed significant differences in the effects of CST across different strokes and distances, providing an important reference for swim-specific training.

As shown in Table 2, the effect of CST varied significantly across different swimming strokes. Among the analyzable stroke types, backstroke showed the greatest improvement (SMD = −0.896, *p* = 0.000), followed by butterfly (SMD = −0.738, *p* = 0.002), while freestyle exhibited a relatively smaller effect size (SMD = −0.599, *p* = 0.000). These differences may be attributed to the biomechanical characteristics of each stroke. The wave-like motion of butterfly requires strong synergy among trunk muscles [24], while backstroke depends on core muscles to maintain balance against water resistance [25]. In this study, the effect of CST on butterfly (SMD = − 0.738) may be attributed to enhanced spinal stability and improved trunk flexion–extension control. Likewise, improvement in backstroke may result from CST-induced strengthening of the transversus abdominis and multifidus muscles, which help maintain a horizontal body position in water [60]. Although the effect size for freestyle (SMD = − 0.599) was significant, it was lower than that of other strokes, possibly due to its unique technical demands. Freestyle propulsion relies primarily on the upper limb stroke [61], while the core mainly contributes to maintaining a streamlined body and reducing lateral sway [25].

However, it is important to note that although the effect size for the breaststroke subgroup was greater than that of the backstroke subgroup, it was based on only two studies (k = 2), which limits the strength of the evidence. Therefore, the findings related to breaststroke technique are presented as descriptive only. Evidence suggests that breaststroke relies particularly on core stability. Its technical execution requires a high degree of trunk coordination during the kick and arm strokes, along with hip joint flexibility and dynamic core stability to maintain a streamlined posture [22]. Studies have demonstrated that arm-leg coordination is crucial in breaststroke [62], and core muscle training enhances power transfer between the trunk and the limbs [63]. In the breaststroke subgroup presented in this study, CST may have improved synchronisation between the kick and arm stroke by enhancing trunk rotational control and hip stability, resulting in a significant time reduction in the 50 m breaststroke. This finding is supported by Seifert et al., who reported that in breaststroke, in-phase coordination time decreases with increasing speed [64]. High-level swimmers adapt by reducing glide time, implying a greater need for arm-leg coordination [64]. Conversely, a weak core can cause energy leakage, leading to a weaker kick and reduced overall power output [65]. Although the findings suggest that CST may yield greater benefits in stroke types with higher technical demands, further high-quality studies with larger sample sizes are needed to confirm this trend.

Subgroup analyses showed that CST had the greatest improvement effect on the 50 m event (SMD = − 0.759, *p* = 0.000), followed by the 100 m (SMD = − 0.608, *p* = 0.000). These results reflect the physiological mechanism of distance-specific adaptation. The 50 m swim is a short-duration, high-intensity exercise dominated by anaerobic metabolism, with performance largely dependent on neuromuscular coordination and explosive power [66]. Core training enhances strength primarily through neural adaptations, such as faster neural activation, improved motor unit synchronization, increased neural recruitment patterns, and reduced inhibitory reflexes [67, 68]. These adaptations are particularly beneficial for sprint swimmers [68]. CST may improve power chain transmission efficiency by enhancing the rapid contractility of core muscles, thereby improving short-term athletic performance. Additionally, Weston et al. found that 12 weeks of core training significantly improved 50 m freestyle performance in adolescent swimmers, likely due to enhanced core function and neuromuscular adaptation [26]. The 100 m swim relies on a combination of anaerobic and aerobic metabolism. CST may improve endurance via two mechanisms: (1) reducing trunk sway, thereby lowering energy expenditure, and (2) enhancing respiratory muscle function (e.g., diaphragm), which increases oxygen uptake efficiency [69]. In the present study, the effect size for the 100 m freestyle (SMD = − 0.432) did not reach statistical significance (*p* = 0.075), though the trend suggests a meaningful effect, possibly limited by small sample size. Hibbs et al. noted that most existing studies are short-term (6–8 weeks), and evidence linking core training directly to athletic performance remains limited [67]. They recommend longer-term, targeted studies to validate CST benefits in specific disciplines, such as long-distance swimming [67]. Similarly, most training weeks in the present study lasted 6–8 weeks, which may have limited the full expression of training effects. In addition, longer-distance swimming events (e.g., 400 m and 800 m) were not included in this study. Therefore, future research should continue to investigate the effects of CST on longer competitive distances.

### Subgroup analysis related to dose parameters

Training parameters—such as weeks, frequency and session duration—are key moderating variables that influence the effects of CST. Subgroup analyses in the present study revealed that training weeks, weekly frequency, and single-session duration were all significant moderators of improvements in swimming performance.

Table 2 shows that the effect size for the 8-week training cycle (SMD = −0.732, *p* = 0.000), although only marginally higher than the 6-week (SMD = −0.617, *p* = 0.052), was not statistically significant for the 6-week. This finding aligns with the physiological principles of motor adaptation. A 6-week training period may induce only neuroadaptive changes—such as improved motor unit recruitment efficiency—but may be insufficient to trigger structural adaptations like muscle fiber hypertrophy [57, 70]. For example, Karpinski et al. found that 6 weeks of CST led to modest improvements in swimming performance, despite increased activation of core muscle groups [28]. In the present study, the relatively small effect size for the 6-week intervention (SMD = − 0.617) may be due to its insufficient duration. An 8-week training weeks is often considered optimal for achieving concurrent improvements in core muscle strength and endurance. In this study, the 8-week training intervention demonstrated a significant effect size (SMD = − 0.732) with low heterogeneity (I^2^ = 18%), potentially indicating an effective balance between physiological adaptation and fatigue accumulation. Additionally, the 8-week training may have enhanced the oxidative capacity of slow-twitch muscle fibers, thereby improving metabolic efficiency during long-distance swimming [71].

Subgroup analyses revealed that training ≤ 3 sessions per week (SMD = −0.778, *p* = 0.000) was more effective than > 3 sessions per week (SMD = −0.537, *p* = 0.001). This finding has important implications for the design of training programmes. A training frequency of ≤ 3 sessions per week may reduce the risk of overtraining while allowing sufficient time for muscular recovery following overload. Ashok et al. reported that muscle fatigue significantly reduces both static and dynamic balance, as well as core strength [72]. In the present study, the effect size for > 3 sessions per week (SMD = − 0.537), although statistically significant, was lower than that for the ≤ 3 sessions group, possibly due to cumulative fatigue.

Effect sizes were greater for single training sessions lasting > 30 to ≤ 60 min (SMD = − 0.731, *p* = 0.000) compared to sessions ≤ 30 min (SMD = − 0.620, *p* = 0.000). This difference may be attributed to the comprehensiveness of the training content. Sessions ≤ 30 min may not allow sufficient time to deliver multidimensional stimulation of the core musculature (e.g., strength, endurance, dynamic stability). For example, Behm noted that shorter training sessions tend to focus primarily on strength, often neglecting endurance and neuromuscular control, thereby limiting overall adaptation [2]. A session duration of > 30 to ≤ 60 min allows for the inclusion of diverse training elements (e.g., Swiss ball balance, suspension training, dynamic trunk rotation), which can effectively activate various functional units of the core musculature [73]. In the present study, this duration yielded a larger effect size, supporting the 'multidimensional stimulation' hypothesis. Furthermore, longer sessions may have facilitated adaptive remodeling of muscle fibers through the accumulation of metabolic stress [74].

Although load intensity was not directly analyzed in this study, existing literature suggests that moderate intensity (60–80% 1RM) is likely optimal for swimming-specific CST. High-intensity training (> 80% 1RM) may elevate injury risk, while low-intensity training (< 60% 1RM) may fail to elicit sufficient neuromuscular adaptations [75]. Additionally, Cormie et al. found that core strength training with moderate loads significantly improved swimmers' start and turn efficiency, though it yielded limited improvements in absolute strength [76].

Additionally, most of the studies included in this review were conducted on adolescent participants (14 out of 15), which may limit the generalisability of the findings to adults due to age-related physiological differences. With natural ageing, collagen synthesis may decrease [77], motor unit recruitment patterns change [78], and the risk of overuse injuries increases during traditional strength training. These changes underscore the need for age-specific studies to establish optimised CST protocols for adults. Therefore, future research should compare the effects of CST across different swimmer age groups.

### Limitations

There are several limitations to this study. 1. Limitations of sample characteristics: The majority of the included studies (13 out of 14) recruited adolescents as participants, which limits the generalizability of the findings to adult swimmers and recreational athletes. Additionally, the breaststroke subgroup included only two original studies, and was therefore analyzed descriptively in this review. As a result, this study lacks sufficient evidence on breaststroke and does not comprehensively cover multiple swimming strokes. 2. Sources of heterogeneity: Although overall heterogeneity was reduced through influence analysis, a moderate degree of residual heterogeneity remained. This may be attributed to variations in the core training protocols applied across studies. Core training is not a standardized or homogeneous intervention; it may encompass core strength, core stability, core endurance, or various combinations of these elements. The specific type, intensity, and combination of exercises used—whether in isolation or synergistically—may exert different effects on athletes’ performance outcomes. 3. Despite our reinterpretation of the PEDro scoring criteria, it must be acknowledged that the studies included in this review generally exhibited a high risk of bias with respect to blinding procedures. 4. Limitations in assessing the dose–response relationship: Most interventions lasted 6 to 8 weeks, and there was a lack of studies investigating longer-term protocols (> 8 weeks). As a result, it was not possible to assess the prolonged effects of CST on swimming performance or its contribution to structural adaptations such as muscle fiber remodeling. Moreover, few studies reported the intensity of training loads, which limited the ability to conduct a detailed analysis of the dose–response relationship. 5. Unanalyzed interaction effects: Most outcome measures were based on freestyle performance, while fewer studies focused on other strokes (e.g., breaststroke, backstroke, butterfly). Moreover, potential interaction effects—such as between gender and stroke type, or between age and training duration—were not systematically examined due to insufficient stratified data.

## Conclusions

This systematic review and meta-analysis demonstrates that CST is a highly effective supplementary intervention for improving swimming performance. The findings provide robust evidence that CST yields a significant, moderate overall improvement in performance (SMD = −0.71) compared to control conditions. The benefits are particularly pronounced in short-distance, anaerobic events such as the 50 m sprint (SMD = −0.759). Among strokes, CST appears to be most effective for backstroke (SMD = −0.896) and butterfly (SMD = −0.738). Regarding training design, an optimal protocol appears to consist of an 8-week intervention, administered in sessions of > 30 to ≤ 60 min, at a frequency of ≤ 3 sessions per week. Future research should focus on establishing standardized CST protocols and investigating its long-term effects and physiological adaptation mechanisms.

## Supplementary Information

Supplementary Material 1.

Supplementary Material 2.

Supplementary Material 3.

Supplementary Material 4.

## Data Availability

All the data used in this study came from publicly available databases.

## Abbreviations

Bac: Backstroke
Bre: Breaststroke
But: Butterfly
CI: Confidence Interval
CS: Core Stability
CST: Core Stability Training
Fre: Freestyle
Med: Medley stroke
SMD: Standardized mean difference
IV: Inverse Variance
PEDro: Physiotherapy Evidence Database
PRISMA: Preferred Reporting Items for Systematic Reviews and Meta-Analyses
PROSPERO: International Prospective Register of Systematic Reviews

## References

[1] Kibler WB, Press J, Sciascia A. The role of core stability in athletic function[J]. Sports Med. 2006;36:189–98. 16526831 10.2165/00007256-200636030-00001

[2] Behm DG, Drinkwater EJ, Willardson JM, et al. The use of instability to train the core musculature[J]. Appl Physiol Nutr Metab. 2010;35(1):91–108. 20130672 10.1139/H09-127

[3] Chaari F, Boyas S, Rebai H, et al. Effectiveness of 12-week core stability training on postural balance in soccer players with groin pain: a single-blind randomized controlled pilot study. Sports Health. 2024. 10.1177/19417381241259988. 39066655 10.1177/19417381241259988 PMC12059599

[4] Ayhan C, Unal E, Yakut Y. Core stabilisation reduces compensatory movement patterns in patients with injury to the arm: a randomized controlled trial[J]. Clin Rehabil. 2014;28(1):36–47. 23823711 10.1177/0269215513492443

[5] Stuber KJ, Bruno P, Sajko S, et al. Core stability exercises for low back pain in athletes: a systematic review of the literature[J]. Clin J Sport Med. 2014;24(6):448–56. 24662572 10.1097/JSM.0000000000000081

[6] Xueqiang W, Jiejiao Z, et al. Effect of core stability training on patients with chronic low back pain[J]. HealthMED. 2012;6(3):754–9.

[7] Chang WD, Lin HY, Lai PT. Core strength training for patients with chronic low back pain[J]. J Phys Ther Sci. 2015;27(3):619–22. 25931693 10.1589/jpts.27.619 PMC4395677

[8] Luo S, Soh KG, Soh KL, et al. Effect of core training on skill performance among athletes: a systematic review. Front Physiol. 2022;13:915259. 35755428 10.3389/fphys.2022.915259 PMC9227831

[9] Yao W. Impacts of core training on athletes’performance in long-distance running[J]. Revista Brasileira de Medicina do Esporte, 2022;29:e2022_0374.

[10] Ding W, Li J, Zhu C. Core training impacts running athletes’ physical capacity. Rev Bras Med Esporte. 2022;29:e2022_0295.

[11] Abt JP, Smoliga JM, Brick MJ, et al. Relationship between cycling mechanics and core stability[J]. J Strength Cond Res. 2007;21(4):1300–4. 18076271 10.1519/R-21846.1

[12] Liu Q, Zhu C, Huang Q. Effects of sling exercise on the core endurance and performance of basketball players. Rev Bras Med Esporte. 2022;29:e2021_0013.

[13] Luo S, Soh KG, Zhao Y, et al. Effect of core training on athletic and skill performance of basketball players: a systematic review. PLoS One. 2023;18(6):e0287379. 37347733 10.1371/journal.pone.0287379 PMC10286970

[14] Luo S, Soh KG, Zhang L, Zhai X, Sunardi J, Gao Y, et al. Effect of core training on skill-related physical fitness performance among soccer players: a systematic review. Front Public Health. 2023;10:1046456. 10.3389/fpubh.2022.1046456. 36684974 10.3389/fpubh.2022.1046456 PMC9850239

[15] Tsai Y, Chia C, Lee P, Lin L, Kuo Y. Landing kinematics, sports performance, and isokinetic strength in adolescent male volleyball athletes: influence of core training. J Sport Rehabil. 2020;29(1):65–72. 10.1123/jsr.2018-0015. 30526235 10.1123/jsr.2018-0015

[16] Ma S, Soh KG, Japar SB, Liu C, Luo S, Mai Y, et al. Effect of core strength training on the badminton player’s performance: A systematic review & meta-analysis. PloS One. 2024;19(6):e0305116. 10.1371/journal.pone.0305116. 38865415 10.1371/journal.pone.0305116 PMC11168634

[17] Rodríguez-Perea Á, Reyes-Ferrada W, Jerez-Mayorga D, et al. Core training and performance: a systematic review with meta-analysis[J]. Biol Sport. 2023;40(4):975–92. 37867742 10.5114/biolsport.2023.123319 PMC10588579

[18] Saeterbakken AH, Stien N, Andersen V, et al. The effects of trunk muscle training on physical fitness and sport-specific performance in young and adult athletes: a systematic review and meta-analysis[J]. Sports Med. 2022;52(7):1599–622. 35061213 10.1007/s40279-021-01637-0 PMC9213339

[19] Dong K, Yu T, Chun B. Effects of core training on sport-specific performance of athletes: a meta-analysis of randomized controlled trials[J]. Behav Sci. 2023;13(2):148. 36829378 10.3390/bs13020148 PMC9952339

[20] Toussaint HM, Beek PJ. Biomechanics of competitive front crawl swimming[J]. Sports Med. 1992;13:8–24. 1553457 10.2165/00007256-199213010-00002

[21] Patil D, Salian SC, Yardi S. The effect of core strengthening on performance of young competitive swimmers. Int J Sci Res. 2014;3(6):2470–7.

[22] Sanders RH, Cappaert JM, Pease DL. Wave characteristics of Olympic breaststroke swimmers[J]. J Appl Biomech. 1998;14(1):40–51.

[23] Willardson JM. Core stability training: applications to sports conditioning programs[J]. J Strength Cond Res. 2007;21(3):979–85. 17685697 10.1519/R-20255.1

[24] Chollet D, Seifert L, Boulesteix L, et al. Arm to leg coordination in elite butterfly swimmers[J]. Int J Sports Med. 2006;27(04):322–9. 16572376 10.1055/s-2005-865658

[25] Psycharakis SG, Sanders RH. Body roll in swimming: a review[J]. J Sports Sci. 2010;28(3):229–36. 20131140 10.1080/02640410903508847

[26] Weston M, Hibbs AE, Thompson KG, Spears LR. Isolated core training improves sprint performance in national-level junior swimmers. Int J Sports Physiol Perform. 2015;10:204–10. 25025936 10.1123/ijspp.2013-0488

[27] Eskiyecek CG, Gul M, Uludag B, Gul GK. The effect of 8-week core exercises applied to 10–12 age male swimmers on swimming performance. Int J Appl Exerc Physiol. 2020;9:213–20.

[28] J. Karpinski et al., The effects of a 6-week core exercises on swimming performance of national level swimmers. Plos One. 2020;15:1–12. 10.1371/journal.pone.0227394 PMC7458297 32866148

[29] Ji MY, Yoon JH, Song KJ, Oh JK. Effect of Dry-Land Core Training on Physical Fitness and Swimming Performance in Adolescent Elite Swimmers. Iranian journal of public health. 2021;50(3):540–549. 10.18502/ijph.v50i3.5595. 10.18502/ijph.v50i3.5595 PMC8214624 34178801

[30] A. Khiyami, S. Nuhmani, R. Joseph, T. S. Abualait, Q. Muaidi. Efficacy of core training in swimming performance and neuromuscular parameters of young swimmers: a randomised control trial. J Clin Med. 2022;11:3198. 10.3390/jcm11113198 PMC9181058 35683586

[31] Scibek, JS. The Effect of Core Stabilization Training on Function Performance in Swimming. Eugene, Or.: Kinesiology Publications, University of Oregon. 1999. OCLC: 1430477129.

[32] Kwok, W. Y., So, B. C. L., Psycharakis, S., & Ng, S. S. M. (Under review). The Effect of the 8-week Core Muscle Training in Swimming Time, Swimming Propulsive Force and Core Muscle Activity Among Swimmers: A Randomized Controlled Trial. European J Sport Sci. 10.52082/jssm.2025.755 PMC12590209 41210095

[33] Hepsert S, Özdemir T, Kılıç Y. Çocuklarda 6 Haftalık Core Egzersizin Bazı Psikomotorve 50 Metre Serbest Stil Yüzme Derecelerine Etkisi[J]. Turkish Studies-Social Sciences, 202217(1):89–96.

[34] Rodríguez S, León-Prieto C, Jaime MFR, et al. Meta-Analysis of the Effects of Core Stability Training on 50-Meter Freestyle Performance in Men and Women[J]. Revista de Investigación e Innovación en Ciencias de la Salud. 2025;7(1):1–14.

[35] Rodríguez S, León-Prieto C, Rodríguez-Jaime M F, et al. Effects of core stability training on swimmers' specific performance: A systematic review with meta-analysis[J]. Journal of Bodywork and Movement Therapies, 2025;42:1063–1072. 10.1016/j.jbmt.2025.03.005 40325637

[36] Page M J, McKenzie J E, Bossuyt P M, et al. The PRISMA 2020 statement: an updated guideline for reporting systematic reviews[J]. BMJ, 2021;372:1–9. 10.1136/bmj.n71 PMC8005924 33782057

[37] Maher CG, Sherrington C, Herbert RD, et al. Reliability of the PEDro scale for rating quality of randomized controlled trials[J]. Phys Ther. 2003;83(8):713–21. 12882612

[38] Moseley AM, Rahman P, Wells GA, et al. Agreement between the Cochrane risk of bias tool and Physiotherapy Evidence Database (PEDro) scale: a meta-epidemiological study of randomized controlled trials of physical therapy interventions. PLoS One. 2019;14(9):e0222770. 31536575 10.1371/journal.pone.0222770 PMC6752782

[39] Kümmel J, Kramer A, Giboin LS, et al. Specificity of balance training in healthy individuals: a systematic review and meta-analysis[J]. Sports Med. 2016;46:1261–71. 26993132 10.1007/s40279-016-0515-z

[40] Liberati A, Altman D G, Tetzlaff J, et al. The PRISMA statement for reporting systematic reviews and meta-analyses of studies that evaluate healthcare interventions: explanation and elaboration[J]. BMJ. 2009;339:1–27. 10.1136/bmj.b2700 PMC2714672 19622552

[41] Higgins J P T, Thompson S G, Deeks J J, et al. Measuring inconsistency in meta-analyses[J]. BMJ. 2003;327(7414):557–560. 10.1136/bmj.327.7414.557 PMC192859 12958120

[42] Cohen J. Statistical power analysis for the behavioral sciences. 2nd ed. Hillsdale: Lawrence Erlbaum; 1988.

[43] Özdoğru, K. yaş grubu erkek yüzücülerde 8 haftalık dinamik kor antrenmanının bazı motorik özellikler ile 100 m karışık stil yüzme performansına etkisi. Yüksek Lisans Tezi, İstanbul Gelişim Üniversitesi, Sağlık Bilimleri Enstitüsü, Antrenörlük Eğitimi Anabilim Dalı, Hareket ve Antrenman Bilimleri Bilim Dalı, İstanbul. 2018:10–12.

[44] Gönener A, Demirci D, Gönener U, et al. 13–15 YAŞ GRUBU ERKEK YÜZÜCÜLERDE 8 HAFTALIK CORE ANTRENMANININ SIRT ÜSTÜ STİLİ 100 M PERFORMANSINA ETKİSİ[J]. Sportif Bakış: Spor ve Eğitim Bilimleri Dergisi, 2017;4(S1): 29–37.

[45] Sedaghati P, Saki F, Sarlak P. The impact of specific core stability training on the sports performance of teenage competitive swimmers. J Rafsanjan Univ Med Sci. 2018;17(4):305–18.

[46] Marani IN, Subarkah A, Octrialin V. The effectiveness of core stability exercises on increasing core muscle strength for junior swimming athletes. Int J Hum Mov Sports Sci. 2020;8:22–8.

[47] Zarei M, Hovanloo F, Ramin N. The effect of core stability exercises with Swiss ball on the performance of sub-elite adolescent swimmers. Stud Sport Med. 2019;10(24):17–28.

[48] Sarikaya M, Salih O. The Effect of 8-Weekly Core Training on 50 Meters Attached Swimming Technique in Fibers of 10–12 Age Group[J]. Int J Soc Humanit Sci Res (JSHSR). 2019;6(46):4152–6.

[49] Mayda Y, Ozdal M, Bilgiç M, et al. The chronic effect of core training on different swimming skills performance in youth swimmers[J]. Eur J Phys Educ Sport Sci. 2024;12(1):1–13.

[50] M. Gül, İ. Alagöz, G. K. Gül, Effect of core stabilization training applied to 10-13 age swimmers on the swimming time and some motoric characteristics. European Journal of Physical Education and Sport Science. 2020;6(1).

[51] Gencer YG. Effects of 8-week core exercises on free style swimming performance of female swimmers aged 9–12[j]. Asian J Educ Train. 2018;4(3):182–5.

[52] Kurt SĆ, Ibis S, Aktug ZB, et al. The effect of core training on swimmers’ functional movement screen scores and sport performances[J]. JTRM in Kinesiology. 2023;9:1–6.

[53] Darchini M, Darzabi T, MofradMoghadam M, et al. The effect of a 6-week core stability training program on the stroke index and front crawl record of male swimmers[j]. J Sport Biomech. 2019;5(2):124–33.

[54] Brown KA, Patel DR, Darmawan D. Participation in sports in relation to adolescent growth and development. Transl Pediatr. 2017;6(3):150–9. 10.21037/tp.2017.04.03. 28795005 10.21037/tp.2017.04.03 PMC5532200

[55] Roemmich JN, Rogol AD. Physiology of growth and development: its relationship to performance in the young athlete. Clin Sports Med. 1995;14:483. 7553919

[56] Ferreira ML, Smeets RJEM, Kamper SJ, et al. Can we explain heterogeneity among randomized clinical trials of exercise for chronic back pain? A meta-regression analysis of randomized controlled trials[J]. Phys Ther. 2010;90(10):1383–403. 20671101 10.2522/ptj.20090332

[57] Schoenfeld BJ, Ogborn D, Krieger JW. Dose-response relationship between weekly resistance training volume and increases in muscle mass: a systematic review and meta-analysis[J]. J Sports Sci. 2017;35(11):1073–82. 27433992 10.1080/02640414.2016.1210197

[58] Yaprak Y, Küçükkubaş N. Gender-related differences on physical fitness parameters after core training exercises: a comparative study[J]. Prog Nutr. 2020;22(3):e2020028–e2020028.

[59] Inbar O, Chia M. Development of maximal anaerobic performance: an old issue revisited[J]. The Young Athlete, 2008;27:27-38.

[60] Hsu C, Krabak B, Cunningham B, et al. Swimming anatomy and lower back injuries in competitive swimmers: a narrative review. Sports Health. 2024;16(6):971–81. 38262981 10.1177/19417381231225213 PMC11531034

[61] Sharp RICKL, Troup JOHNP, Costill DAVIDL. Relationship between power and sprint freestyle swimming. Med Sci Sports Exerc. 1982;14(1):53–6. 7070258 10.1249/00005768-198201000-00010

[62] Takagi H, Sugimoto S, Nishijima N, et al. Swimming: differences in stroke phases, arm-leg coordination and velocity fluctuation due to event, gender and performance level in breaststroke[J]. Sports Biomech. 2004;3(1):15–27. 15079985 10.1080/14763140408522827

[63] Dingley AA, Pyne DB, Youngson J, Burkett B. Effectiveness of a dry-land resistance training program on strength, power, and swimming performance in paralympic swimmers. J Strength Cond Res. 2015;29:619–26. 25226306 10.1519/JSC.0000000000000684

[64] Seifert L, Leblanc H, Chollet D, et al. Inter-limb coordination in swimming: effect of speed and skill level[J]. Hum Mov Sci. 2010;29(1):103–13. 19945188 10.1016/j.humov.2009.05.003

[65] Fig G. Strength training for swimmers: training the core. Strength Cond J. 2005;27(2):40–2.

[66] Hung, C.-H.; Su, C.-H.; Wang, D. The role of high-intensity interval training (HIIT) in neuromuscular adaptations: implications for strength and power development—a review. Preprints 2025, 2025031163. 10.20944/preprints202503.1163.v1. 10.3390/life15040657 PMC12028608 40283211

[67] Hibbs AE, Thompson KG, French D, et al. Optimizing performance by improving core stability and core strength[J]. Sports Med. 2008;38:995–1008. 19026017 10.2165/00007256-200838120-00004

[68] Reed CA, Ford KR, Myer GD, et al. The effects of isolated and integrated ’core stability’training on athletic performance measures: a systematic review[J]. Sports Med. 2012;42:697–706. 22784233 10.2165/11633450-000000000-00000 PMC4166601

[69] Lomax M, McCONNELL A. Inspiratory muscle fatigue in swimmers after a single 200 m swim[J]. J Sports Sci. 2003;21(8):659–64. 12875316 10.1080/0264041031000101999

[70] Folland JP, Williams AG. Morphological and neurological contributions to increased strength[J]. Sports Med. 2007;37:145–68. 17241104 10.2165/00007256-200737020-00004

[71] Hawley JA. Adaptations of skeletal muscle to prolonged, intense endurance training[J]. Clin Exp Pharmacol Physiol. 2002;29(3):218–22. 11906487 10.1046/j.1440-1681.2002.03623.x

[72] Ashok S, Parag S. Effect of induced muscular fatigue on balance and core strength in normal individuals[J]. Int Editorial Advisory Board. 2014;8(3):187.

[73] McGill S. Core training: evidence translating to better performance and injury prevention[J]. Strength Cond J. 2010;32(3):33–46.

[74] Schoenfeld BJ. The mechanisms of muscle hypertrophy and their application to resistance training[J]. J Strength Cond Res. 2010;24(10):2857–72. 20847704 10.1519/JSC.0b013e3181e840f3

[75] Suchomel TJ, Nimphius S, Bellon CR, et al. The importance of muscular strength: training considerations[J]. Sports Med. 2018;48:765–85. 29372481 10.1007/s40279-018-0862-z

[76] Cormie P, McGuigan MR, Newton RU. Develo** maximal neuromuscular power: part 2—training considerations for improving maximal power production[J]. Sports Med. 2011;41:125–46. 21244105 10.2165/11538500-000000000-00000

[77] Ackerman JE, Bah I, Jonason JH, et al. Aging does not alter tendon mechanical properties during homeostasis, but does impair flexor tendon healing[J]. J Orthop Res. 2017;35(12):2716–24. 28419543 10.1002/jor.23580 PMC5645212

[78] Cruz-Jentoft AJ, Baeyens JP, Bauer JM, et al. Sarcopenia: European consensus on definition and diagnosis: report of the European Working Group on Sarcopenia in Older People[J]. Age Ageing. 2010;39(4):412–23. 20392703 10.1093/ageing/afq034 PMC2886201

