# Correction: The effects of core stability training on swimming performance in youth swimmers: a systematic review and meta‑analysis

**DOI:** 10.1186/s13102-026-01588-x

**Published:** 2026-02-18

**Authors:** Shunfang Liu, Jinming Dai, Pengpeng Gou, Menglong Lin

**Affiliations:** 1 School of Physical Education and Sports Science, South China Normal University, Guangzhou, China; 2 Program in Global Exercise Science, Arts & Sports College, Inha University, Incheon, South Korea; 3 Faculty of Sport and Leisure, Guangdong Ocean University, Zhanjiang, China

**Correction: BMC Sports Sci Med Rehabil 17**,** 327 (2025)**

10.1186/s13102-025-01366-1

Following publication of the original article [1], the authors identified an error in the author name of Jinming Dai.

The incorrect author name is: Jingling Dai.

The correct author name is: Jinming Dai.

The author group has been updated above and the original article [1] has been corrected.
