# Supplementary material for: The effects of core stability training on swimming performance in youth swimmers: a systematic review and meta-analysis
Source: BMC Sports Sci Med Rehabil. 2025 Nov 11;17:327. doi: 10.1186/s13102-025-01366-1 (PMC12606982; doi:10.1186/s13102-025-01366-1)
Supplement: Supplementary file 2 — Supplementary Material 2. [file 13102_2025_1366_MOESM2_ESM.docx]

**Appendix B Database Search Terms**

**Web of Science**

TS=(Core Training OR Core Strength Training OR Core Stability Training OR Core Endurance Training OR Core Muscles Training OR Core Exercises) AND TS=(Swimming Exercise OR Swimming Performance OR Swimming)

**ScienceDirect**

('Core Training' OR 'Core Strength Training' OR 'Core Stability Training' OR 'Core Endurance Training' OR 'Core Muscles Training' OR 'Core Exercises') AND ('Swimming Exercise' OR 'Swimming Performance' OR Swimming)

**Proquest**

subject('Core Training' OR 'Core Strength Training' OR 'Core Stability Training' OR 'Core Endurance Training' OR 'Core Muscles Training' OR 'Core Exercises') AND subject('Swimming Exercise' OR 'Swimming Performance' OR Swimming)

**PubMed**

((((((Core Training[MeSH Major Topic]) OR (Core Strength Training[MeSH Major Topic])) OR (Core Stability Training[MeSH Major Topic])) OR (Core Endurance Training[MeSH Major Topic])) OR (Core Muscles Training[MeSH Major Topic])) OR (Core Exercises[MeSH Major Topic])) AND (((Swimming Exercise[MeSH Major Topic]) OR (Swimming Performance[MeSH Major Topic])) OR (Swimming[MeSH Major Topic]))

**Embase**

('Core Training' OR 'Core Strength Training' OR 'Core Stability Training' OR 'Core Endurance Training' OR 'Core Muscles Training' OR 'Core Exercises') AND ('Swimming Exercise' OR 'Swimming Performance' OR Swimming)

**Scopus**

ALL ( core AND training OR core AND strength AND training OR core AND stability AND training OR core AND endurance AND training OR core AND muscles AND training OR core AND exercises ) AND ALL ( swimming AND exercise OR swimming AND performance OR swimming )

**Cochrane Library**

ID Search Hits

#1 Core Training 4786

#2 Core Strength Training 1513

#3 Core Stability Training 855

#4 Core Endurance Training 530

#5 Core Muscles Training 700

#6 Core Exercises 2258

#7 #1 OR #2 OR #3 OR #4 OR #5 OR #6 5661

#8 Swimming Exercise 1067

#9 Swimming Performance 684

#10 Swimming 1691

#11 #8 OR #9 OR #10 1691

#12 #7 AND #11 117

**SPORTDiscus**

('Core Training' OR 'Core Strength Training' OR 'Core Stability Training' OR 'Core Endurance Training' OR 'Core Muscles Training' OR 'Core Exercises') AND ('Swimming Exercise' OR 'Swimming Performance' OR Swimming)
