# Supplementary material for: The effects of core stability training on swimming performance in youth swimmers: a systematic review and meta-analysis
Source: BMC Sports Sci Med Rehabil. 2025 Nov 11;17:327. doi: 10.1186/s13102-025-01366-1 (PMC12606982; doi:10.1186/s13102-025-01366-1)
Supplement: Supplementary file 3 — Supplementary Material 3. [file 13102_2025_1366_MOESM3_ESM.docx]

**Appendix C PEDro Evidence Assessment**

**Author(s): Shunfang Liu**
**Date:** 2025-03-26

| NO. | Author | Year | PEDro scale items (1 point if there are any of the following conditions, 0 points if there are none) | | | | | | | |
| --- | --- | --- | --- | --- | --- | --- | --- | --- | --- | --- |
|  |  |  |  |  |  |  |  |  |  |  |
|  |  |  | random allocation | concealed allocation | baseline similarity | completeness  of follow-up | intention-to-treat analysis | between-group statistical comparisons | point measurements and variability | Total points |
| 1 | Ahmad Khiyami | 2022 | 1 | 1 | 1 | 0 | 1 | 1 | 1 | 6 |
| 2 | Canan Gülbin Eskıyecek | 2020 | 1 | 1 | 0 | 1 | 1 | 1 | 1 | 6 |
| 3 | Jakub Karpiński | 2020 | 1 | 1 | 0 | 1 | 1 | 1 | 1 | 6 |
| 4 | Kaan Özdoğru | 2018 | 1 | 1 | 1 | 1 | 1 | 1 | 1 | 7 |
| 5 | Ahmet Gönener | 2017 | 1 | 1 | 1 | 1 | 1 | 1 | 1 | 7 |
| 6 | P. Sedaghati | 2018 | 1 | 1 | 1 | 1 | 1 | 1 | 1 | 7 |
| 7 | Kwok Wan Yu | 2024 | 1 | 1 | 1 | 0 | 1 | 1 | 1 | 6 |
| 8 | Ika Novitaria Marani | 2020 | 1 | 1 | 0 | 1 | 1 | 1 | 1 | 6 |
| 9 | Dnyanesh Patil | 2014 | 1 | 1 | 1 | 1 | 1 | 1 | 1 | 7 |
| 10 | Mostafa Zarei | 2017 | 1 | 1 | 1 | 1 | 1 | 1 | 1 | 7 |
| 11 | Mucahit SARIKAYA | 2019 | 1 | 1 | 1 | 1 | 1 | 1 | 1 | 7 |
| 12 | Yaşar Mayda | 2025 | 1 | 1 | 0 | 1 | 1 | 1 | 1 | 6 |
| 13 | Mine Gül | 2020 | 1 | 1 | 0 | 1 | 1 | 1 | 1 | 6 |
| 14 | Yıldırım Gökhan GENCER | 2018 | 0 | 0 | 1 | 1 | 1 | 1 | 1 | 5 |
| 15 | Songül KURT | 2023 | 0 | 0 | 0 | 1 | 1 | 1 | 1 | 4 |
| 16 | M Darchini | 2019 | 1 | 1 | 0 | 1 | 1 | 1 | 1 | 6 |
