# Supplementary material for: The effects of core stability training on swimming performance in youth swimmers: a systematic review and meta-analysis
Source: BMC Sports Sci Med Rehabil. 2025 Nov 11;17:327. doi: 10.1186/s13102-025-01366-1 (PMC12606982; doi:10.1186/s13102-025-01366-1)
Supplement: Supplementary file 4 — Supplementary Material 4. [file 13102_2025_1366_MOESM4_ESM.docx]

**Appendix D Correlation Chart**

**Forest plot**

**
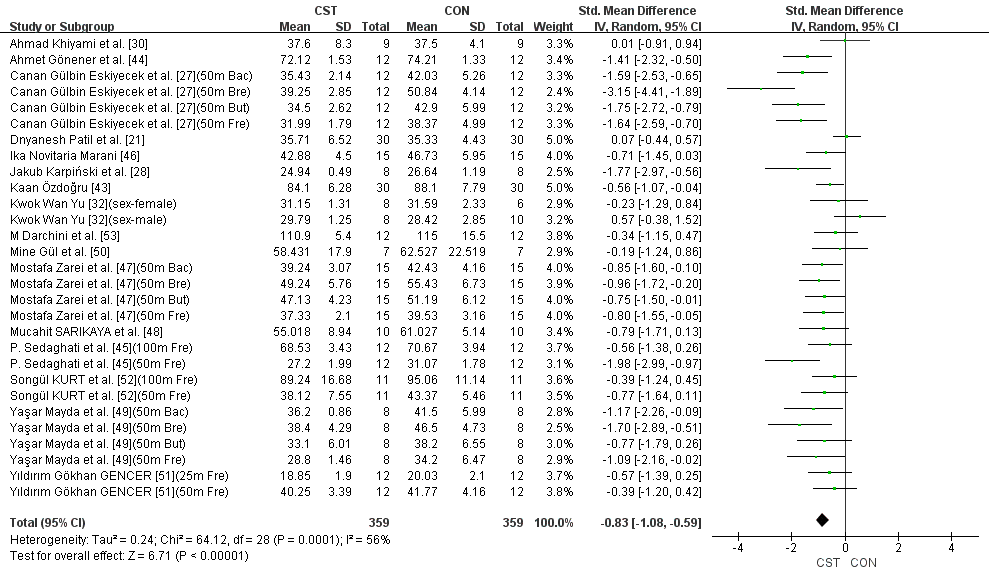
**

Before removing heterogeneous data

**
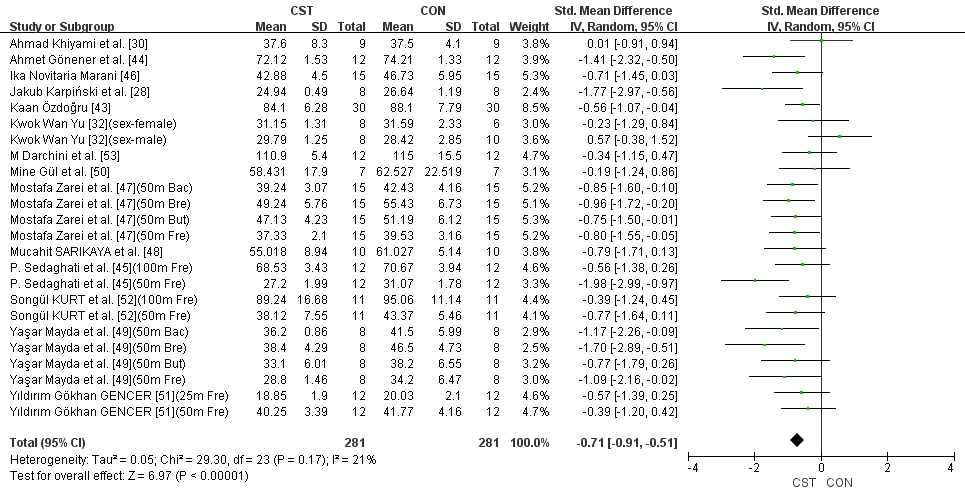
**

After removing heterogeneous data

**Funnel chart**

**
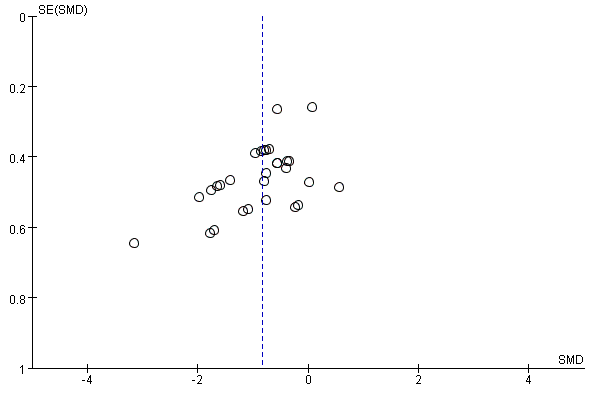
**

Before removing heterogeneous data


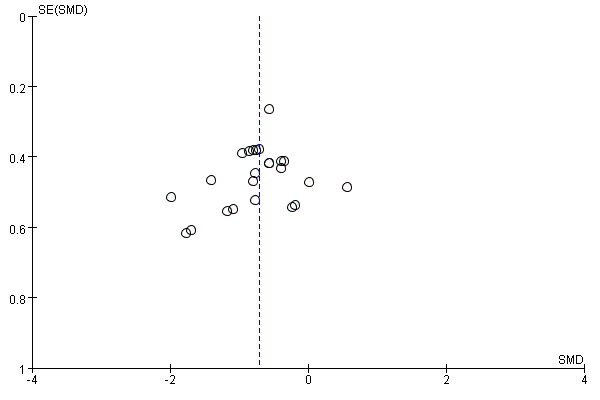


After removing heterogeneous data

**Influence analysis**

**
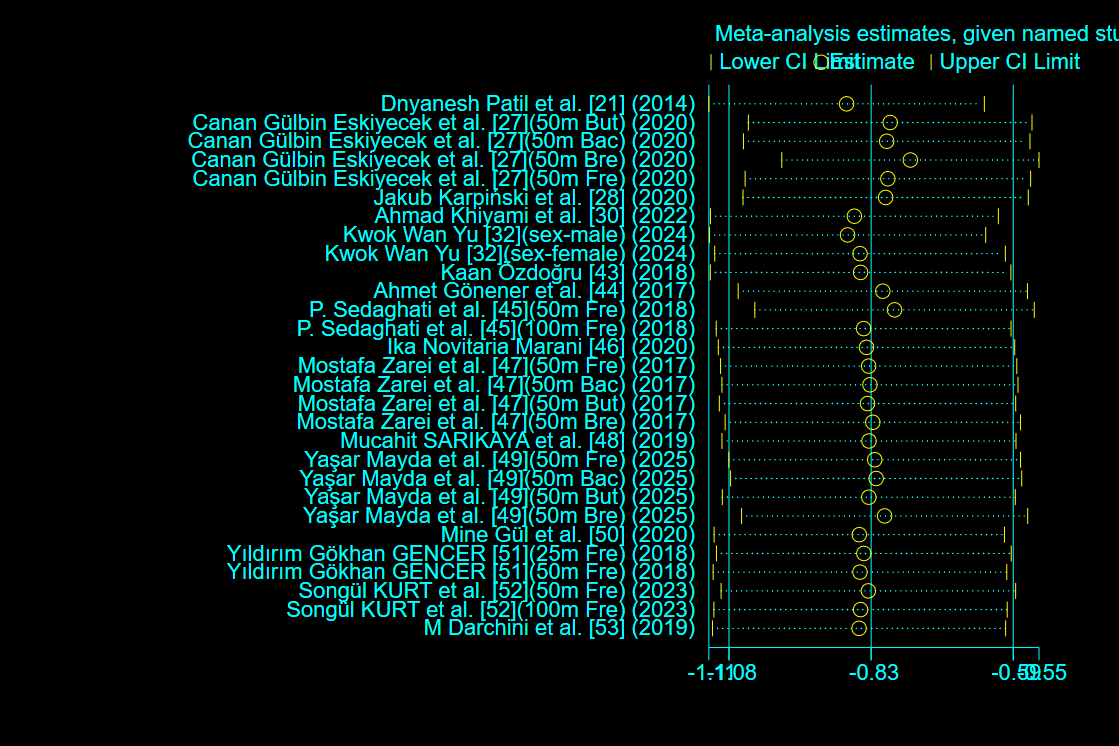
**

Before removing heterogeneous data


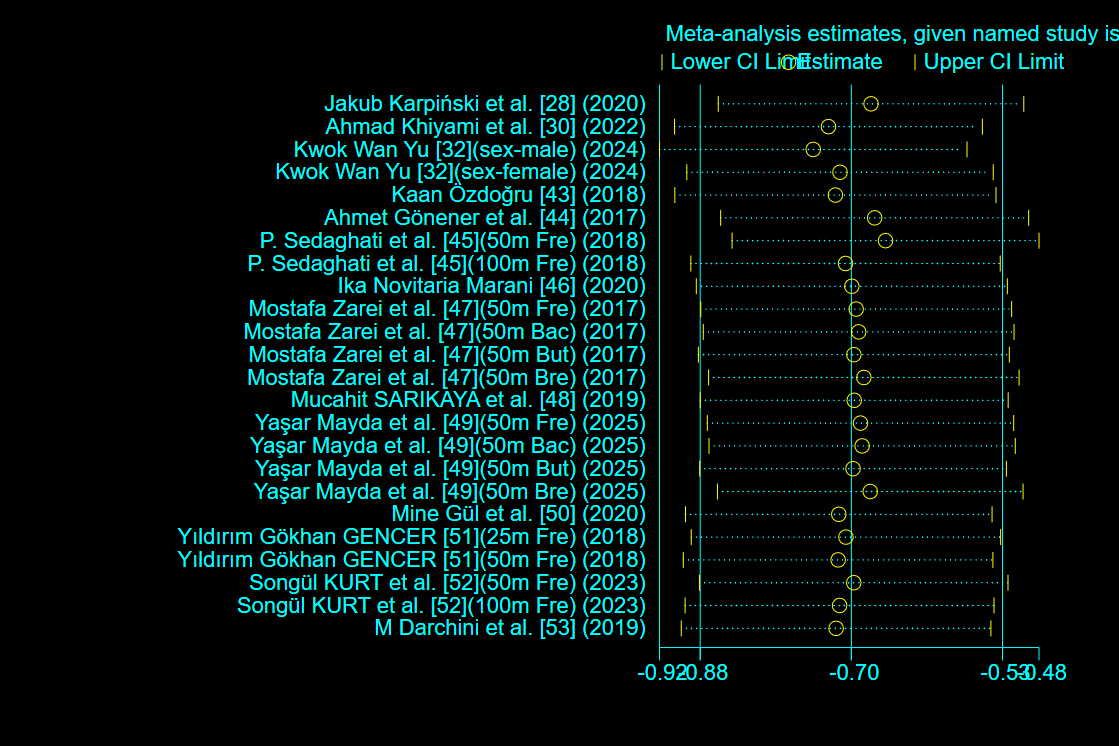


After removing heterogeneous data

**Egger’ test**


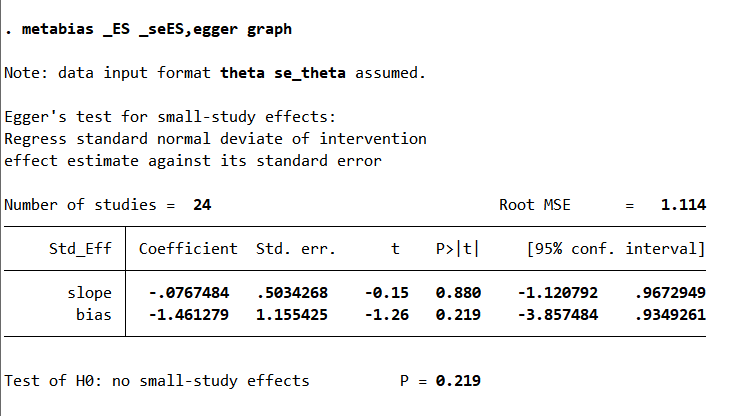


**Subgroup analysis diagram**

**
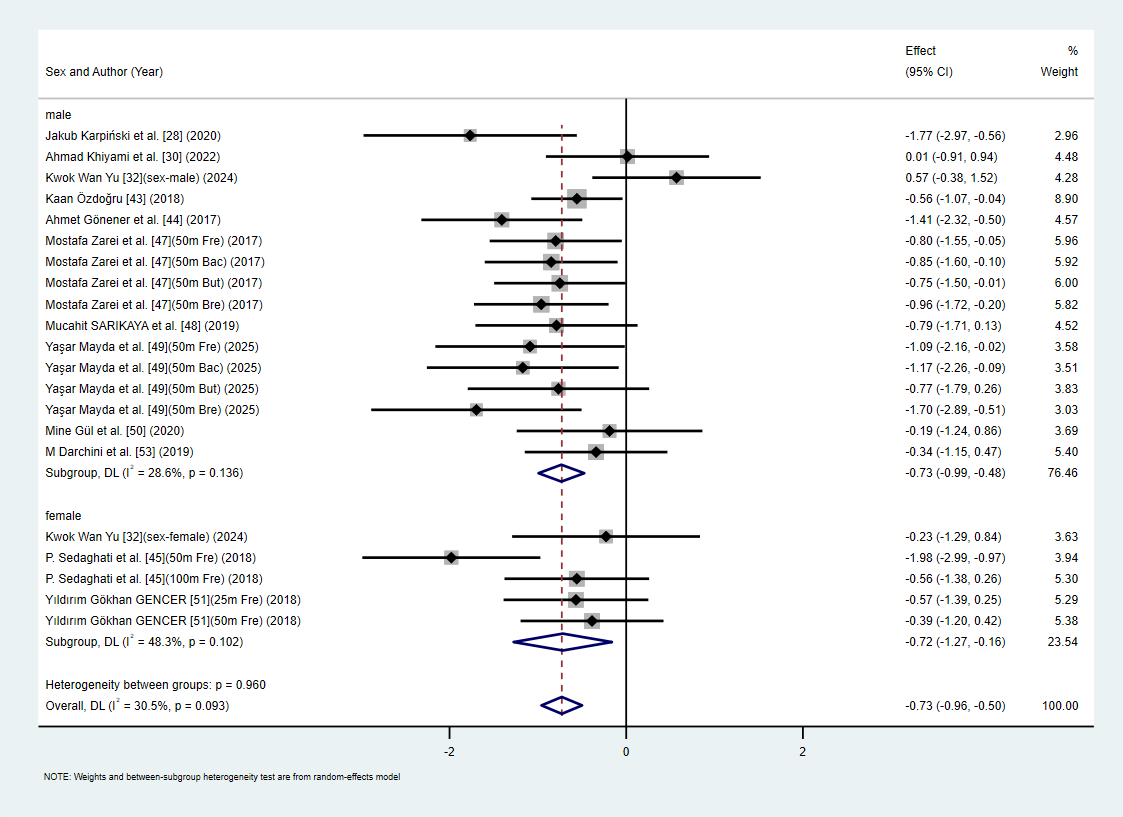
**

Gender-based grouping


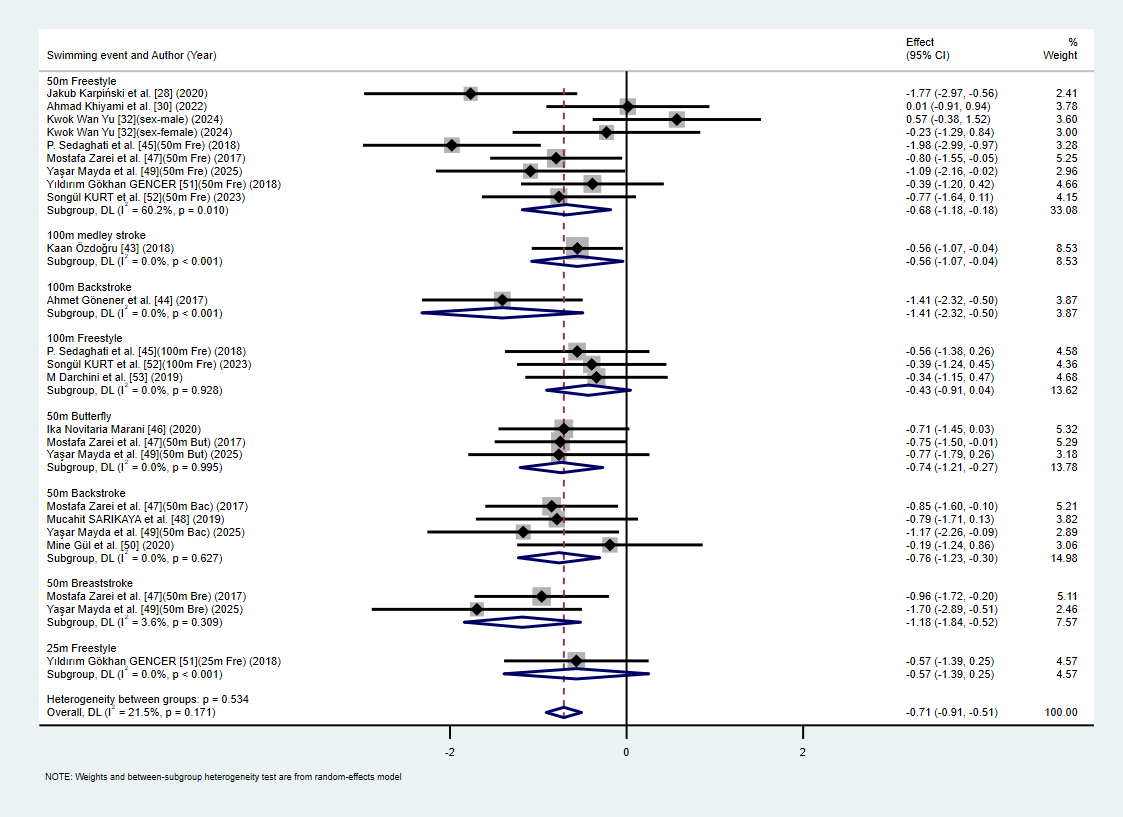


Groups based on swimming events


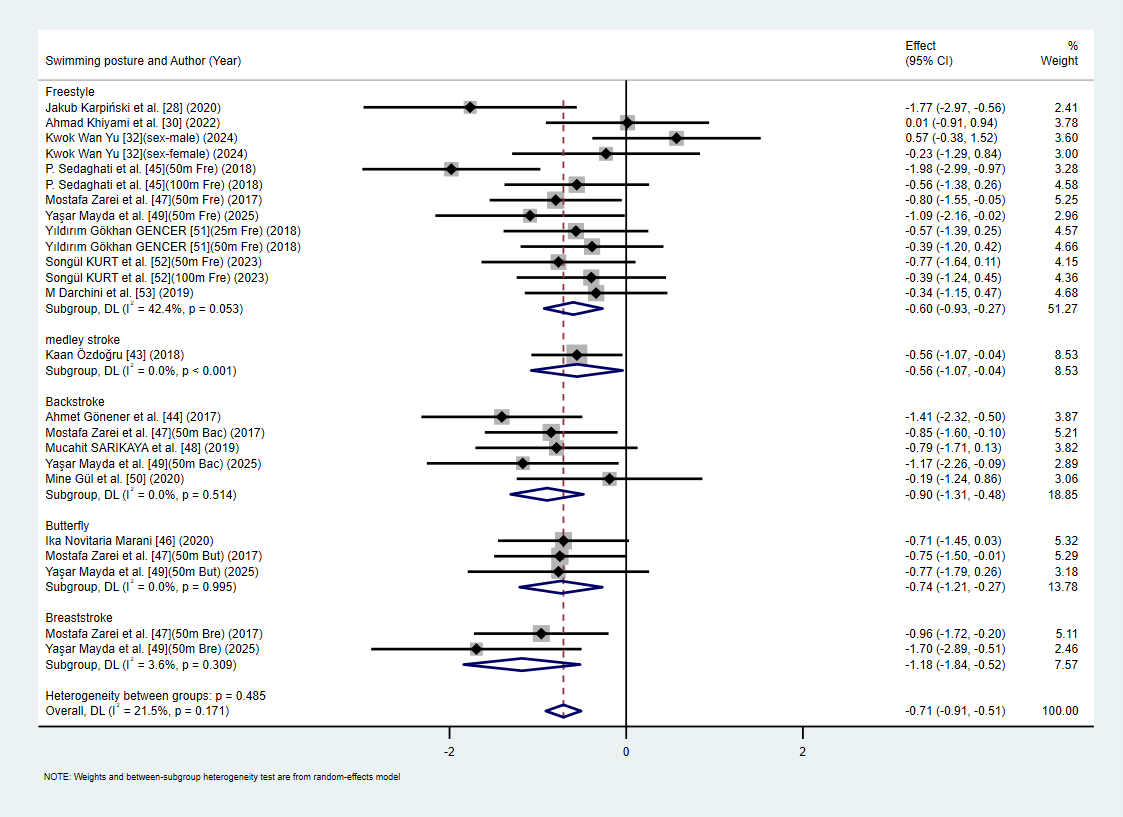


Groups based on swimming posture


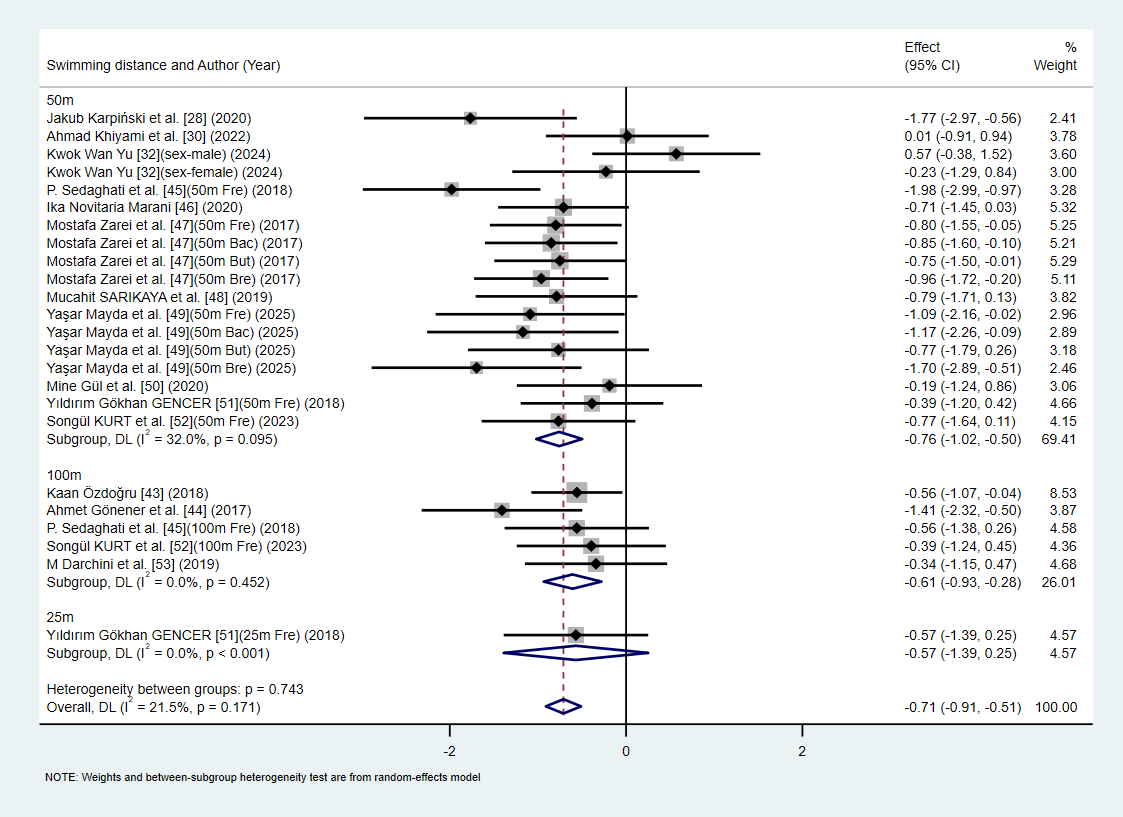


Groups based on swimming distance


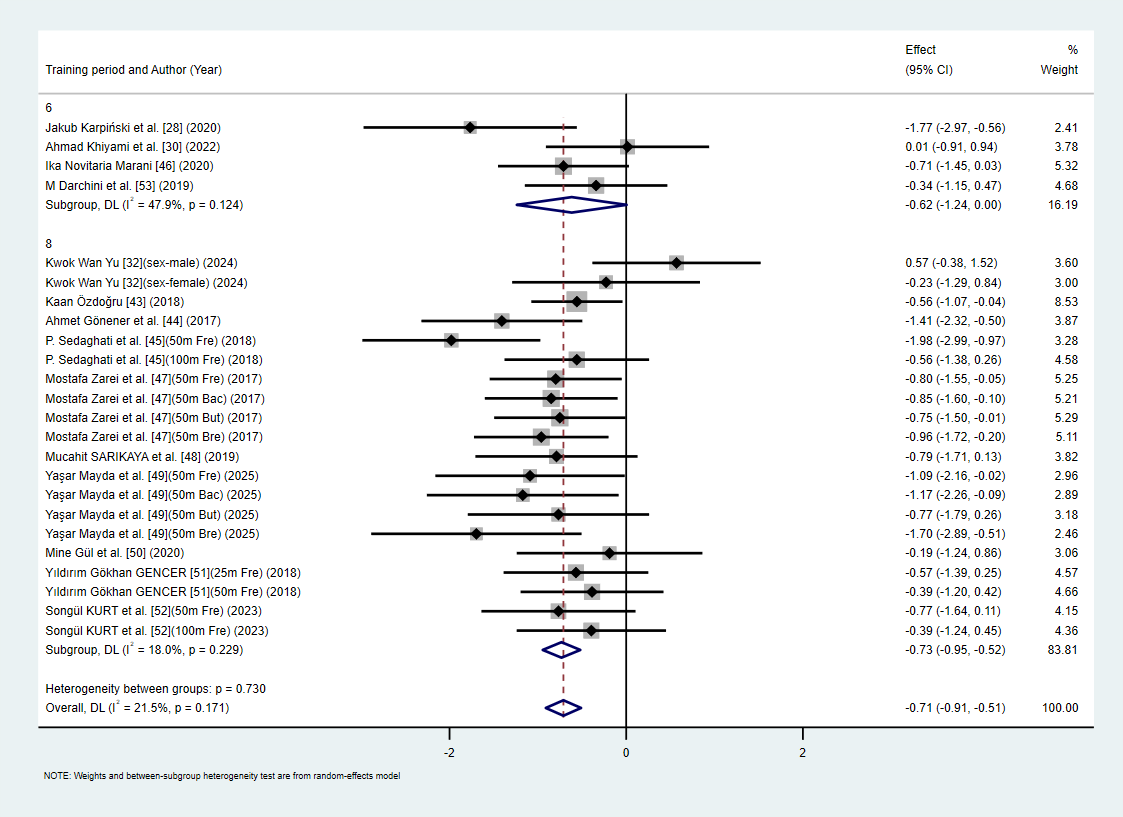


Period-based grouping


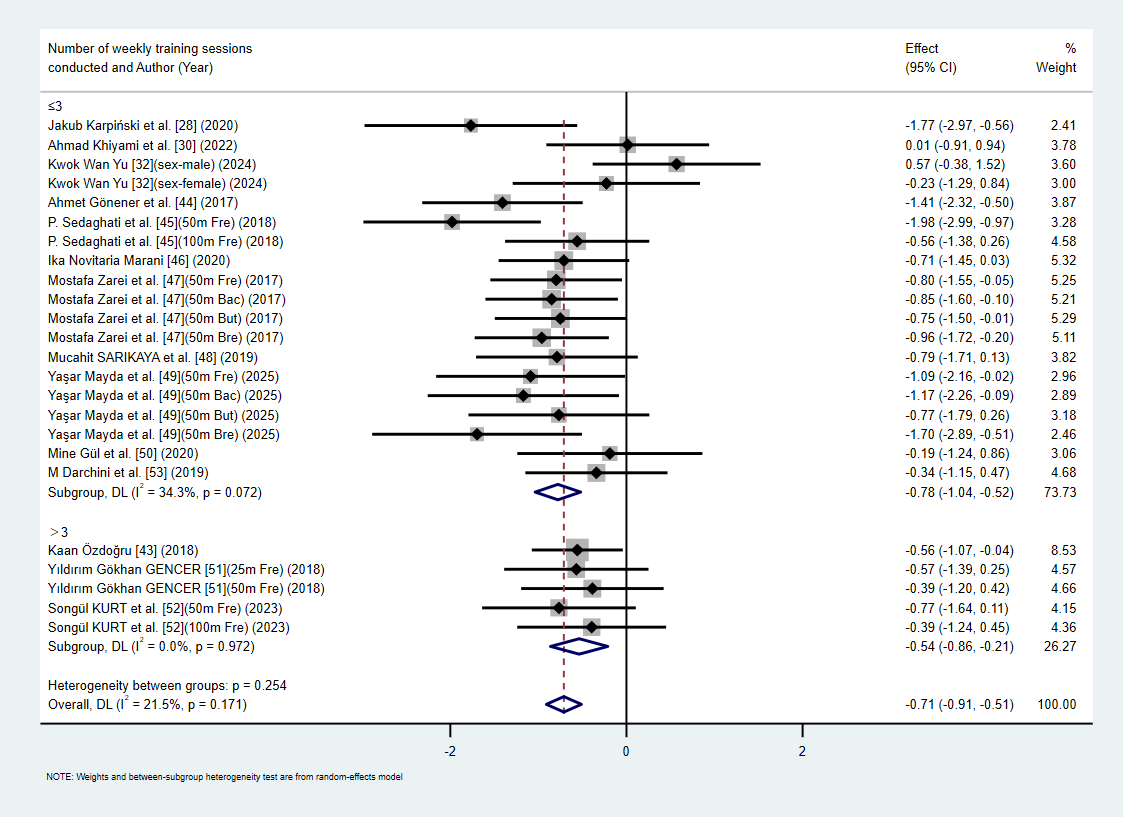


Frequency-based grouping


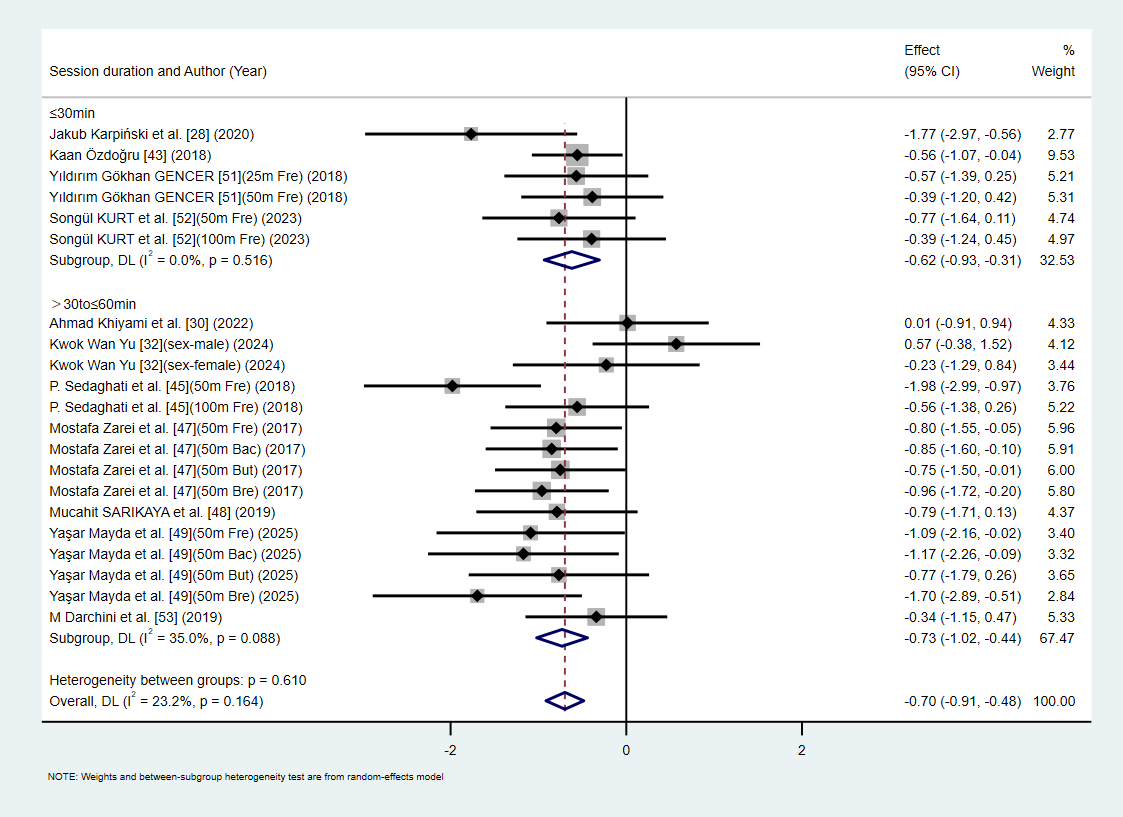


Grouping based on a single duration
